# Zero-Point-Energy Driven Isotopic Exchange of the [H_3_O]^−^ anion Probed by Mid-Infrared Action Spectroscopy

**DOI:** 10.1021/jacs.4c05543

**Published:** 2024-07-25

**Authors:** Dennis F. Dinu, Milan Ončák, Sven Thorwirth, Klaus R. Liedl, Sandra Brünken, Stephan Schlemmer, Pavol Jusko

**Affiliations:** † Institute of Materials Chemistry, TU Wien, Getreidemarkt 9/165, 1060 Vienna, Austria; ‡ Department of General, Inorganic and Theoretical Chemistry, Universität Innsbruck, Innrain 80/82, 6020 Innsbruck, Austria; § Institut für Ionenphysik und Angewandte Physik, Universität Innsbruck, Technikerstrasse 25, 6020 Innsbruck, Austria; ∥ I. Physikalisches Institut, Universität zu Köln, Zülpicher Str. 77, 50937 Koln̈, Germany; ⊥ Radboud University, FELIX Laboratory, Institute for Molecules and Materials, Toernooiveld 7, 6525 ED Nijmegen, The Netherlands; # Max Planck Institute for Extraterrestrial Physics, Giessenbachstrasse 1, 85748 Garching, Germany

## Abstract

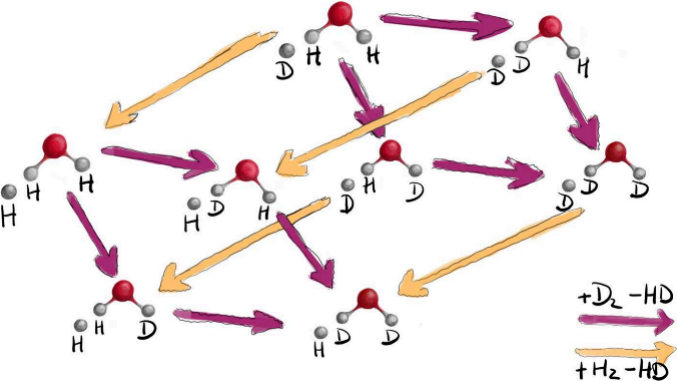

We present the first observation of vibrational transitions in the [H_3_O]^−^ anion, an intermediate in the anion–molecule reaction of water, H_2_O, and hydride, H^–^, using a laser-induced isotopic H/D exchange reaction action spectroscopy scheme applied to anions. The observed bands are assigned as the fundamental and first overtone of the H_2_O–H^–^ vibrational stretching mode, based on anharmonic calculations within the vibrational perturbation theory and vibrational configuration interaction. Although the D_2_O·D^–^ species has the lowest energy, our experiments confirm the D_2_O·H^–^ isotope to be a sink of the H/D exchange reaction. *Ab initio* calculations corroborate that the formation of D_2_O·H^–^ is favored, as the zero-point-energy difference is larger between D_2_ and H_2_ than between D_2_O·H^–^ and D_2_O·D^–^.

## Introduction

**Figure 1:**
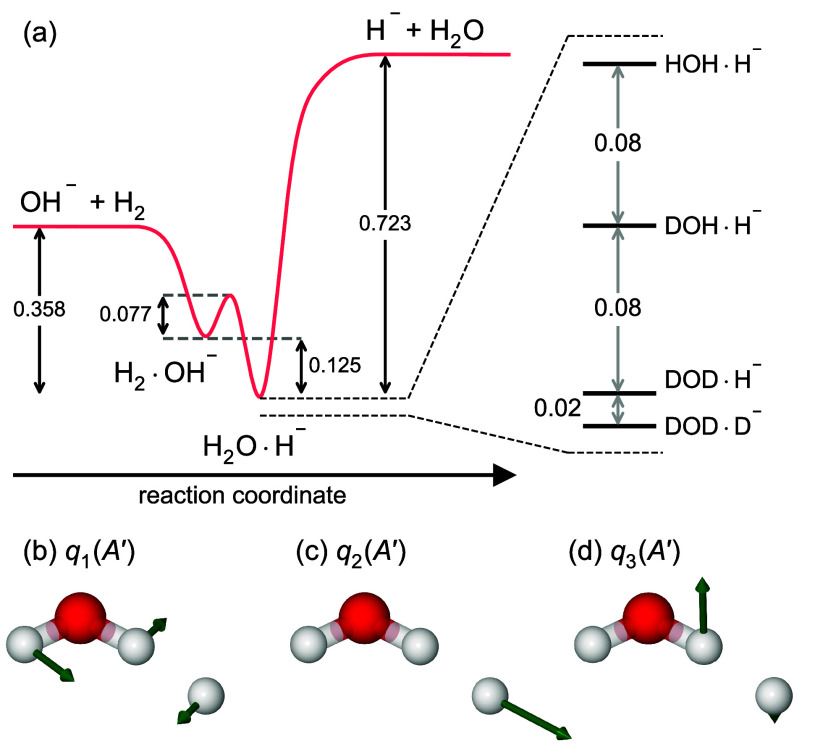
(a) Energy separation (eV) between different systems with 3H, O, and e^–^,^9^ together with zero-point energies of D-isotopes of the most stable configuration HOH·H^–^ (harmonic approximation). (b–d) Three lowest vibrational modes of HOH·H^–^.

The binding of the water molecule with a charged species, i.e., a proton H^+^, an electron e^–^, or a hydride H^–^, is one of the crucial subjects in fundamental physical chemistry.^1^ While the binding of H^+^ to the water molecule H_2_O has been extensively explored and is often a benchmark in newly developed experimental and theoretical studies (recent example: a large-scale spectroscopic line list of H_3_O^+^ in the ExoMol database^2^), the [H_3_O]^−^ anionic species remains essentially uncharted, as it is much more difficult to stabilize. In its pyramidal structure like that of the H_3_O^+^ cation,^3^ the [H_3_O]^−^ anion is a hypothetical double Rydberg anion.^4^ However, the most stable conformation of [H_3_O]^−^ is a hydride H^–^ bound to a water H_2_O molecule, with the hydride acting as a strong base,^5^ which is likely to deprotonate the water molecule. In this regard, the properties of the [H_3_O]^−^ anion are an intimate part of the prototypical anion–molecule reaction
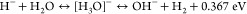
1first reported as early as 1957 by Muschlitz,^6^ and has intrigued theoreticians and experimentalists ever since. Reaction 1 is depicted along the reaction coordinate with deuterated isotopes of the lowest energy intermediate H_2_O ·H^–^ in Figure 1a. The stable hydrated-hydride H_2_O·H^–^ intermediate was first proposed in 1968 based on *ab initio* calculations,^7^ with the first experimental observation only being reported in 1982.^8^ H_2_O ·H^–^ has six normal modes of vibration, of which the three with the lowest frequencies are depicted in Figure 1b–d, but experimental vibrational spectra are missing to date.

During the 1980s, for the [H_3_O]^−^ system, the following has been experimentally established. (a) The migration of H^–^ is nonregioselective, with 50% of HOD·H^–^ and 50% of DOH ·H^–^ being produced in a Kleingeld–Nibbering reaction of OH^–^ + CH_2_O ↔ HOH ·HCO^–^ → HOH ·H^–^ inside a Fourier transform ion cyclotron resonance mass spectrometer, where the exothermic hydride transfer from HCO^–^ dominates.^10^ (b) E.g., in HO^–^ + D_2_, the deuterium transfer causes a switch of the solvating species from the poorly solvating D_2_ to the better solvating HOD, by using a variable-temperature selected ion flow tube.^11^ (c) HOH·H^–^ is indeed the most stable form of [H_3_O]^−^, based on photoelectron spectroscopy^12^ and by using a tandem mass spectrometer to study the products of OH^–^·H_2_O + H_2_ → HOH·H^–^ + H_2_O.^13^ (d) The dissociation pathway to OH^–^ is always favored (as Figure 1a suggests), by collision-induced dissociation of [H_3_O]^−^.^14^

In 1994, Miller et al. asserted that the HOH·H^–^ cluster is lower in energy than the OH^–^·H_2_ cluster, as water is a better anion ligand than H_2_.^15^ Additionally, multiple *ab initio* calculations in the 1980s and 1990s supported the conclusion that HOH·H^–^ is the most stable structure (cf. refs (16−20) and references therein). In light of the theoretical studies, a collision-induced dissociation experiment confirmed the evidence for a linear OH^–^·H_2_ and a bent HOH·H^–^ structure.^21^

The neutral reaction
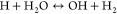
2is connected to reaction 1 by the photodetachment of one electron. Hence, in photoelectron spectroscopy studies of reaction 2, much information about the [H_3_O]^−^ system has been obtained, especially considering the reaction energy surface.^9,12,22,23^

Photoelectron studies of [H_3_O]^−^ from 1995 supported by *ab initio* calculations also revealed that two processes contribute to the spectra: photodetachment from OH^–^·H_2_ and from HOH·H^–^.^22^ Time-dependent wave packet calculations showed that the photodetachment of an electron from OH^–^·H_2_ is dominated by an H_2_ + OH fragment with a small contribution from an HOH + H fragment.^23^

In the 2000s, crossed molecular beams were used to investigate the reactive and nonreactive collisions of OH^–^ + D_2_ proceeding through the OH^–^·D_2_ intermediate.^24^ Using the same technique for the same reaction, Li et al.^25^ discovered that D^+^ transfer from D_2_ to OH^–^ results primarily in the excitation of the H–O–D bending vibrational mode of the molecular product. This proton transfer dynamics (OH^–^ + D_2_ → HOD + D^–^) has been investigated computationally in 2020, providing a global potential energy surface for [H_3_O]^−^.^26^

The interest in [H_3_O]^−^ was reignited in 2014 through cryogenic radio frequency ion trap experiments. In an OH^–^ spectroscopic experiment, it was shown that OH^–^(ν > 0) + H_2_ produces H^–^ and water (eq 1).^27^ This shows the other side of the dissociation channel from [H_3_O]^−^(eq 1), compared to the OH^–^ channel observed by de Lange and Nibbering,^14^ to be also accessible. Although the applied spectroscopic scheme proceeded through an intermediate [H_3_O]^−^ with a mass-to-charge ratio of 19 *m*/*z*, its direct observation was not possible since the energy available is too high for the intermediate to be formed and stabilized in a two-body collision, even for OH^–^ in ν = 0.

In the 2010s, the intermediate [H_3_O]^−^ has also been taken advantage of in a spectroscopic experiment on OD^–^ ^28^ and overtone spectroscopy of OH^–^.^29^ Notably, to the best of our knowledge, the only spectroscopy experiment of [H_3_O]^−^ itself consists of an electron detachment study by Hauser et al.,^30^ where different electron affinities of OH^–^·H_2_ (2.05 eV) and H^–^·H_2_O (1.5 eV) were used to confirm the fact that the [H_3_O]^−^ anion produced in a ternary attachment inside a cryogenic trap is predominantly in the H_2_O·H^–^ form.

In summary, many studies have been devoted to the fundamental [H_3_O]^−^ system, mostly addressing the H^–^ + H_2_O and OH^–^ + H_2_ dissociative channels. However, while the structure of the stable [H_3_O]^−^ complex is thought to have the H_2_O·H^–^ form, vibrational spectra of [H_3_O]^−^ could help to reveal its structure experimentally.

In the present investigation, we show the first experimental observation of vibrational transitions for [H_3_O]^−^ and its isotopologues using an H/D exchange action spectroscopic scheme, laser-induced reactions (LIR), routinely used for cations,^31−33^ in a cryogenic 22 pole rf (radio frequency) ion trap, where the number of ions is too low for conventional infrared absorption spectroscopy. The experimental observations, acquired at the FELion beamline of the free-electron laser facility FELIX,^79^ are supported by anharmonic frequency calculations and calculated reaction energies relying on zero-point energies. The agreement between theoretical predictions and experiments will provide clear evidence for the proposed H_2_O·H^–^ structure of the stable [H_3_O]^−^ complex.

## Results and Discussion

### Spectroscopy of [H_3_O]^−^ Isotopologues

**Figure 2:**
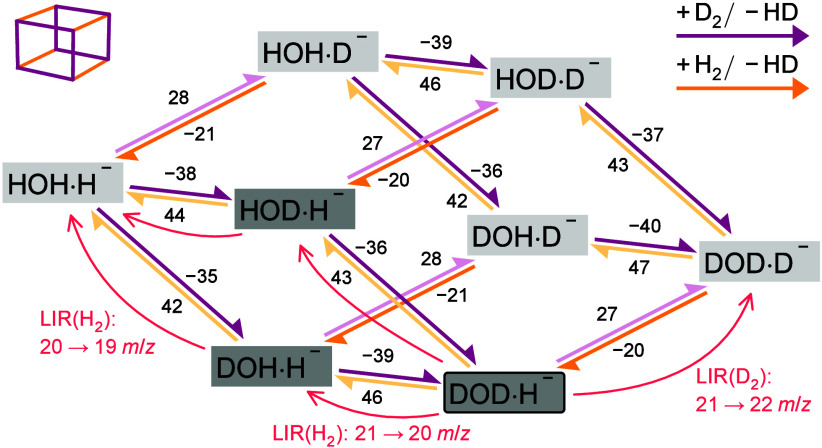
Deuteration (magenta) and hydrogenation (yellow) scheme showing the energy (meV) needed to exchange H_2_ or D_2_ for HD with one of the XOX·X^–^, calculated at the CCSD(T)-F12/VTZ-F12 level of theory. The laser-induced reaction scheme (with the reactant gas in parentheses) and the mass change observed in the experiment are marked with red curved lines. The DOH·H^–^, HOD·H^–^, and DOD·H^–^ species (marked with a dark gray background) exhibit induced reactivity. DOD·H^–^ (marked with a round box) acts as a sink; i.e., all the pathways are exothermic toward it, none out of it.

All possible hydrogen–deuterium exchange reactions between the eight possible XOX·X^–^ species (where X is H or D) in collision with H_2_ and D_2_ are shown in Figure 2. It is instructive to perceive this scheme as a cube made up of XOX·X^–^ species in the corners with the edges representing the reaction arrows. Moving along the edge from left to right always represents deuteration; the opposite direction represents hydrogenation. Correspondingly, moving downward releases energy (exothermic), while moving upward requires energy (endothermic).

If all of the XOX·H^–^ species are seen as one side of the cube, all of the XOX·D^–^ species are on the opposite side. In this notation, one can directly see two characteristics of the hydrogen–deuterium exchange reaction. (a) Switching from the XOX·D^–^ side to the XOX·H^–^ side is always energetically favorable. (b) For both “sides”, the energy decreases from the undeuterated to the deuterated water submolecule due to the zero-point energy difference. These H/D exchanges are expected to occur in the ion trap due to the presence of H_2_ and/or D_2_ and indeed, as many of them are exothermic, can be directly observed as a change in mass of the stored ions as a function of storage time (see the Experimental Section).

**Figure 3:**
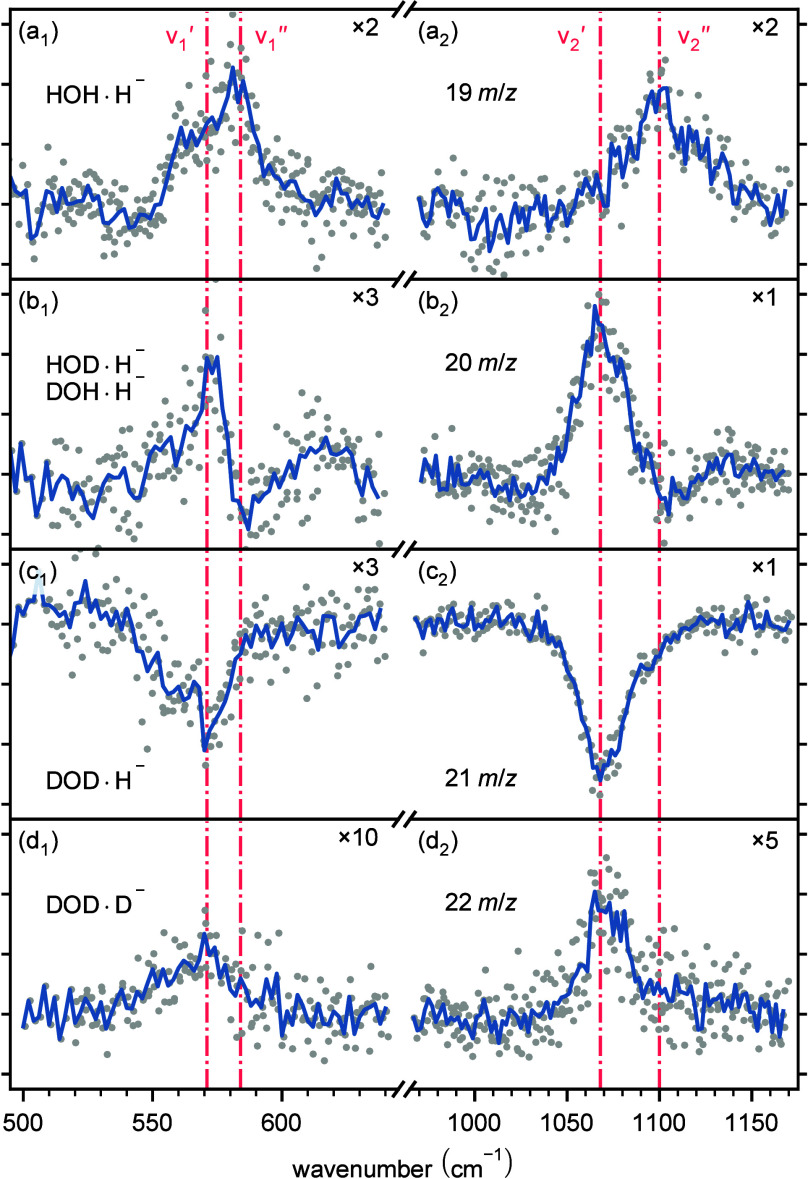
Laser-induced processes on 19–22 *m*/*z*, respectively, showing the change in equilibria induced by the resonantly absorbed photon by DOD·H^–^ (dips in (c_1_, c_2_)) and in HOD·H^–^ and DOH·H^–^ (dips in (b_1_, b_2_)). The signal increase represents the product(s) of the particular LIR reaction (see the text). Red dash-dotted lines represent the band positions ν_1_*′*–ν_2_″ at 571, 584 cm^–1^ and 1068, 1100 cm^–1^. Numbers after the × symbol in the right top corner of each panel represent a scaling factor for the intensity therein.

As the ions are stored in a cryogenic trap, at temperatures <20 K (∼2 meV), with translational and internal energy an order of magnitude lower than reaction endothermicities, the equilibrium between different XOX·X^–^ isotopologues can easily be altered by adding energy into the system, e.g., through resonant IR excitation. This procedure is the basis of our action spectroscopy scheme, where we report the number of stored ions as a function of the tunable laser wavenumber (Figure 3). We identify and depict two active regions where the ion numbers are affected with an overall tuning range between 500 and 1600 cm^–1^. As seen in Figure 3, some species exhibit an increase in numbers, others decrease, and some both.

The following LIR scheme describes the whole process
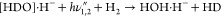
3
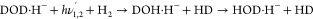
4
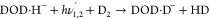
5where ν_1_*′*–ν_2_″ are the wavenumbers where a resonant change of the ion signals is observed, as indicated in Figure 3. The only indication of a decrease of a reactant, i.e., the confirmation that the species is the reactant in the LIR scheme, can be clearly seen in Figure 3c, 21 *m*/*z* (eq 4), and partially, next to a significant increase, in Figure 3b, 22 *m*/*z* (eq 3). At the same time, this significant increase in Figure 3b is caused by the LIR process on Figure 3c, i.e., conversion of 21 → 20 *m*/*z* (product side of eq 4), as well as for Figure 3a, 20 → 19 *m*/*z* (product side of eq 3), and Figure 3d, 21 → 22 *m*/*z* (product side of eq 5). These processes can equally well be identified in Figure 2, where the LIR processes together with reactants (H_2_ for laser-induced hydrogenation, eqs 3 and 4, and D_2_ for laser-induced deuteration, eq 5) are marked with curved upward pointing arrows.

All the LIR processes, together with the observation that DOD·H^–^ can be converted to D_2_O·D^–^ only upon and not without IR activation (see the Experimental Section) are consistent with the *ab initio* calculations, suggesting that DOD·H^–^ is a global sink for the hydrogen–deuterium exchange reactions in the XOX·X^–^ system.

### Spectral Assignment

In the spectra in Figure 3 it is possible to assume HOD·H^–^ and/or DOH·H^–^ for 20 *m*/*z* as optically active isomer(s). For 21 *m*/*z*, it is indisputably DOD·H^–^, as seen from the reaction exo-/endothermicities (see Figure 2). From a comparison to the calculated frequencies (Table 1), it is most likely that the experimentally observed features ν_1_*′* and ν_1_″ arise from *q*_2_(*A′*). This normal mode can be interpreted as the stretching vibration along the dissociation (hydrogen detachment), as shown in Figure 1c. The experimentally observed bands ν_2_*′* and ν_2_″ can be assigned to the first overtone 2*q*_2_(*A′*).

**Table 1:** Anharmonic VPT2 Frequencies in cm^–1^ of the Intermolecular Modes of HOH·H^–^ Calculated at CCSD(T)-F12/aug-cc-pVTZ-F12

	*m*/*z*	*q*_1_(*A′*)	*q*_2_(*A′*)	*q*_3_(*A′*)	2*q*_1_(*A′*)	2*q*_2_(*A′*)	*q*_1_+*q*_2_(*A′*)
HOH·H^–^	19	469.1	602.9	938.8	883.2	1156.9	1045.0
HOD·H^–^	20	431.8	590.1	719.3	818.5	1123.5	996.3
DOH·H^–^	20	394.0	590.4	925.5	756.2	1128.1	962.5
DOD·H^–^	21	371.7	582.1	706.9	711.7	1131.1	924.1
HOH·D^–^	20	424.5	486.6	915.6	820.2	950.8	880.6
HOD·D^–^	21	393.8	458.4	685.6	754.6	895.6	829.1
DOH·D^–^	21	365.5	445.3	902.3	699.5	866.0	798.0
DOD·D^–^	22	343.0	438.5	673.6	658.6	851.9	768.4
LIR	20	584 (ν_1_″)			1100 (ν_2_″)		
LIR	21	571 (ν_1_*′*)			1068 (ν_2_*′*)		

The LIR observed frequencies decrease with decreasing *m*/*z*. That means from 20 to 21 *m*/*z* the frequency decreases from ν_1_″ = 584 cm^–1^ to ν_1_″ = 571 cm^–1^ and from ν_2_″ = 1100 cm^–1^ to ν_2_*′* = 1068 cm^–1^, respectively. The VPT2 calculations, as shown in Table 1, confirm this decrease for the fundamental mode *q*_2_(*A′*), i.e., the frequency decreases from *q*_2_(*A′*) = 590.1/590.4 cm^–1^ (HOD·H^–^/DOH·H^–^) to *q*_2_(*A′*) = 582.1 cm^–1^ (DOD·H^–^). The same VPT2 calculations, however, do not confirm this decrease for the overtone mode 2*q*_2_(*A′*), i.e., the frequency increases from 2*q*_2_(*A′*) = 1123.5/1128.1 cm^–1^ (HOD·H^–^/DOH·H^–^) to *q*_2_(*A′*) = 1131.1 cm^–1^ (DOD·H^–^). These discrepancies are most likely due to limits in the computation, as discussed in the next section.

Given our calculations, the assignment of the observed band at around 1100 cm^–1^ as an overtone is the best-fitting choice. However, this assignment should be approached with care. Our calculated absorption intensities do not predict the strong intensities observed in the experiments. At the same time, the experimental intensities are not true IR absorption intensities but intensities derived from the action scheme. At ν_1_, or 561 cm^–1^, each photon carries approximately 70 meV of energy, which is of similar magnitude to the endothermicities of the H/D exchange reaction in question. A difference in LIR intensities compared to calculated IR intensities at low wavenumbers (below 1000 cm^–1^) has been already observed in a system energetically similar to ours, i.e., in the CH_5_^+^ LIR spectrum recorded using endothermic proton transfer to CO_2_, where the endothermicity is only 31 meV.^34^ Possible explanations for this effect where the calculated IR intensity has been high and the intensity of the respective LIR spectrum was small at around 500 cm^–1^ but comparable at >1000 cm^–1^ has been discussed in some detail.^34^ Following these discussions, we can only speculate that the photon energy can be distributed into all degrees of freedom (vibrational, rotational, translational) of both neutral and anion products of the LIR scheme. We except strong nonlinear behavior for the LIR intensity, i.e., difference in LIR product channel efficiency, including channels leading back to the same reactants in excited states, which are not registered as spectroscopic signals in our experiment, while the photon energy is decreased toward the LIR endothermicity threshold. Note that the photon energy in the overtone transitions ν_2_*′*, ν_2_″ is almost twice as high as in the fundamental ν_1_*′*, ν_1_″.

### Calculated Vibrational Frequencies

The HOH·H^–^ or OH^–^·H_2_ structures represent shallow minima connected by a relatively low barrier (cf. ref (26) and Figure 1). Hence, local expansions of the PES around one of these minima, e.g., harmonic potentials or quartic force fields (QFF), can be problematic, as they neglect the change in the PES curvature close to the barrier.

As expected, calculated harmonic frequencies are too large compared to the experiment (see Table S2 in the Supporting Information). Anharmonic frequencies from perturbation theory (VPT2) agree slightly better with the experiment, considering a HOH·H^–^ minimum (see Table 1). However, considering an OH^–^·H_2_ minimum, VPT2 fails for very low-lying frequencies (see the Supporting Information).

The VPT2 calculated anharmonic frequencies are still sufficient for interpreting the LIR spectra in the present work. They confirm that the experimental spectrum corresponds to the HOH·H^–^ minimum structure and allow reasonable assignment of the experimentally observed frequencies, as discussed above. However, the VPT2 calculations may not accurately predict the overall spectrum, i.e., features that are, for now, experimentally unknown.

More precise theoretical predictions can be expected for a better description of the PES together with variational solutions to the vibrational Schrödinger equation. In this respect, we calculated an *n*-mode PES with up to 4-mode couplings to perform VSCF/VCI calculations for HOH·H^–^, similar to our previous studies on anionic systems (Cl^–^·H_2_,^35^ C_2_N^–^ and C_3_N^–^^36^),

In contrast to QFF, used for VPT2, the *n*-mode PES includes the correct double minimum shape along the normal mode *q*_5_. Our calculations demonstrate that this feature in the PES causes convergence issues in the VCI calculations. The excitations in configurations comprising the mode *q*_5_ must be limited. While these exploratory VCI calculations confirm the interpretation we made from VPT2 for HOH·H^–^, they also show (see the Supporting Information) the necessity for further computations to predict the complete vibrational spectrum.

Such computations may be founded on a global PES, e.g., the fundamental invariant neural network PES by Li et al.^26^ Using this PES to calculate reaction dynamics from quasi-classical trajectory simulations, Li et al. demonstrate reasonable agreement with scattering experiments. However, they merely present harmonic frequencies. It remains to be investigated whether anharmonic frequencies obtained from such a global PES can describe experimentally observed frequencies.

## Conclusions

We presented a first step toward the vibrational spectroscopy and quantitative structure of H_2_O·H^–^ using an action spectroscopic scheme in a cryogenic ion trap. The understanding of the energetics of the H/D exchange, the basis of the applied action process, aided by *ab initio* calculations turned out to be crucial in the development of this LIR probing spectroscopic scheme. We present first experimental information for the binding of H^–^ to water by providing experimental and theoretical frequencies of the stretching vibration in the bond coordinate (fundamental mode and first overtone). We show that the zero point energy between D_2_/H_2_ is surprisingly larger than that between XOX·H^–^/XOX·D^–^.

The vibrational perturbation theory of the second order (VPT2) is sufficient to interpret the experimental LIR spectrum. However, its underlying quartic force field (QFF) remains a crude approximation for the rather floppy HOH·H^–^ structure. Consequently, VPT2 shows nonsystematic deviations from the experiment. Further validation of the accuracy of harmonic and VPT2 frequencies is desirable. As the present investigation shows, probing vibrational transitions for the HOH·H^–^ system by experiment is tedious. While we assigned the *q*_2_(*A′*) fundamental and its first overtone, the other vibrational transitions remain experimentally unobserved. This finding may also indicate that only the excitation of the HOH·H^–^ stretching vibration promotes the D/H transfer reaction, while the excitation of the other modes does not.

From a theoretical point of view, more sophisticated calculations could serve as a future reference. We calculated an *n*-mode PES, which can be considered more accurate than the QFF used in VPT2. However, converging vibrational self-consistent field and configuration interaction (VSCF/VCI) calculations based on this PES were challenging. In the future, theoretical studies using variational calculations for the time-independent Schrödinger equation with a suitable Hamiltonian are required. Alternatively, one may perform multiconfiguration time-dependent Hartree calculations to grasp the dynamics of the reaction.

From an experimental point of view, on the one hand, the presented action spectroscopic scheme should be straightforwardly applicable for the O–H and O–D stretching regions (H_2_O, 3650 cm^–1^; D_2_O, 2670 cm^–1^; HDO, 2720 cm^–1^;^37^ also Tables S4–S11 in the Supporting Information for XOX·H^–^), where readily available high-resolution laser sources could provide the rovibrational resolution, i.e., accurate structural information. On the other hand, the presented LIR scheme is not ideally suited for studies of the pure nondeuterated [H_3_O]^−^, as any attempt to use the endothermic reaction H_2_O·H^–^ + D_2_ → H_2_O·D^–^ + HD as an LIR scheme will inevitably lead to very efficient concurrent water substructure deuteration at the same time. Perhaps novel action techniques based on the coupling of internal (vibrational) excitation to motion in collisions, like leak-out-spectroscopy (LOS),^38^ would have to be applied.

## Methodology

### Experimental Section

All experiments have been conducted in the FELion 22 pole rf cryogenic ion trap setup.^39^ Ions were produced in a Gerlich type storage ion source^40^ using electron bombardment. The main precursor gas was N_2_O, which produces mostly the O^–^ anion, that has enough time to undergo collisions with added precursor gases (H_2_/D_2_). This reaction, unfortunately, leads predominantly to associative detachment, i.e., to H_2_O and e^–^. This process has no “cation” analogue, where the charge is lost toward e^–^, and only a few percent branch toward OH^–^ (17 *m*/*z*) and OD^–^ (18 *m*/*z*) and atomic H/D.^41,42^ It was impossible to produce any of the H_2_HO^–^ isotopes directly in the room-temperature ion source.

**Figure 4:**
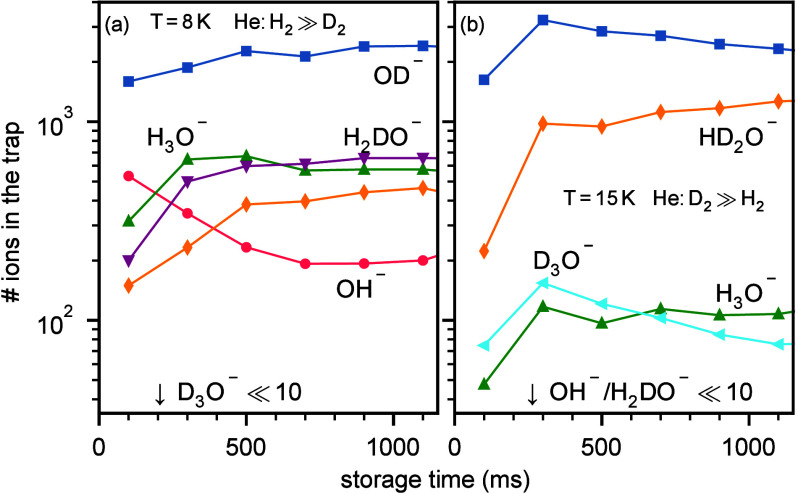
Number of stored ions of characteristic *m*/*z* values as a function of storage time. After the initial intense He pressure is pumped out (hundreds of ms), H/D exchange is the only reaction channel available. (a) Conditions where mostly H_2_ is present in the trap (similar conditions are used for spectroscopy in Figure 3a–c). (b) Conditions where D_2_ is the dominant neutral in the trap (at least 2 orders of magnitude higher than H_2_; similar conditions were used for the spectroscopy in Figure 3d).

Both (and only) masses 17 and 18 *m*/*z* are selected in a first quadrupole mass filter configured for a “two mass band pass” before their injection into the cryogenic ion trap, where they are trapped using an intense He:H_2_ or He:D_2_ (ca. 5:1) pulse. No masses other than those were guided to the trap. We assume the H_2_HO^–^ anion (respectively its deuterated analogues) is created in a ternary collision of the buffer gas helium and neutral H_2_ (respectively D_2_) introduced into the trapping volume. After the initial intense pulse (100–200 ms) is pumped out, the formation of H_2_HO^–^ (19 *m*/*z*) is essentially halted and only H/D exchange can occur inside the trap (see Figure 4). The ions are stored for a predefined storage time from a few milliseconds up to 3 s, mass-analyzed in a second quadrupole mass filter, and consequently detected in a microchannel plate (MCP) detector.

Since we only worked with H, D, and O atoms and ions of 17–22 *m*/*z*, the water anion H_2_O^–^ could be problematic to our measurements, as it has 18 *m*/*z*, exactly the same as for OD^–^. However, it is highly unlikely that we produce this anion. First, according to ATcT^43,80^ one needs approximately 300 kJ mol^–1^ to produce H_2_O^–^ in a reaction of OH^–^ + H_2_, an energy that we do not achieve in our experiment. Second, it was shown that [H_2_O]_*n*_^–^ is efficiently produced only for *n* > 1.^44,45^ Third, H_2_O^–^ was observed with a lifetime of only 4 μs.^46^

For the reaction OH^–^ + D_2_ the H/D exchange is exothermic with a temperature independent reaction rate of *k*_H→D_ ≈ 5 × 10^–10^ cm^3^ s^–1^.^47^ For the back reaction OD^–^ + H_2_, the D/H exchange with the normal-H_2_ (i. e., 3:1 ortho:para H_2_) is endothermic with a very steep positive temperature dependence, essentially reaching 5 × 10^–10^ cm^3^ s^–1^ at 200 K, while playing a negligible role for cold ions at *T* < 20 K with a value of *k*_D→H_ ≈ 10^–12^ cm^3^ s^–1^.^47^ Moreover, the experiment with pure para-H_2_, the spin isomer of H_2_ with zero excess energy, for the hydrogenation of OD^–^ revealed that the rate is indeed lower, down to <10^–14^ cm^3^ s^–1^ below 20 K.^48^ The same nature of the isotope exchange rates has recently been confirmed also for HD exchange with OH^–^^49^ and OD^–^.^50^ Although the absolute values of H/D exchange reaction rates for the [X_3_O]^−^ anion with H_2_/D_2_ play no role in the current study, our experiment assumed and confirms the comparable order of magnitude as for the H/D exchange rates for the [OX]^−^.

Normal hydrogen (deuterium), i.e., 3:1 ortho:para H_2_ (2:1 para:ortho D_2_), as seen at room temperature has been used throughout the experiment. The excess energy of less than 15 meV,^51^ contained in the ortho-H_2_ (even lower for para-D_2_), should not play a significant role in our reaction system, as it is lower than the lowest calculated reaction enthalpy of 21 meV (see Figure 2).

We have to note here that the H/D exchange toward the fully deuterated H_2_HO^–^ did not work as expected from the naïve extension of the OH^–^ + D_2_ and OD^–^ + H_2_ systems. We expected D_2_DO^–^ to be a sink for the H/D exchange, but we could not produce significant D_2_DO^–^ amounts unless very high D_2_ number densities, with almost no H_2_, were continuously injected into the trap, supporting the explanation given in Figure 2 and the *ab initio* calculations.

### Acquisition of LIR Spectra

For the spectroscopic experiment normal-H_2_ mixed with He (ratio 1:2), a typical total number density in the <10^12^ cm^–3^ range (or D_2_ in the case of D_2_O·D^–^ LIR experiment, up to 10^14^ cm^–3^; see Figures 2 and 3), is added into the trap continuously during the whole storage time (typically between 1.6 and 2.6 s). Simultaneously, pulsed free-electron laser light from FEL-2 of FELIX (up to 10 mJ macropulse energy at 10 Hz with fwhm of 0.5% of the center frequency) is used to excite the anions into rovibrational states. The anions collide with the neutral H_2_ (or D_2_) before their radiative de-excitation, which is assumingly on the order of several to tens of milliseconds.

The excited ions, even at a kinetic temperature of 20 K, have total energy ≫200 K, where the hydrogenation reaction rate *k*_D→H_ ≈ 5 × 10^–10^ cm^3^ s^–1^ is comparable to the deuteration reaction rate.^47,48^ Therefore, the excitation caused by the resonant photon absorption changes the equilibrium number of all anions (17–22 *m*/*z*) in the trap. The spectra, i.e., the number of all ions in the trap after the storage (irradiation) time as a function of the wavenumber of the free electron laser, is plotted in Figure 3. Spectra are normalized to the photon power delivered into the trap.

### Computational Details

All the calculations presented here have been performed using the MOLPRO software package,^52−54^ version 2023.1, for anharmonic calculations with the VPT2 program^55^ and VSCF/VCI programs,^56−63^ and PES generations using the XSURF program.^56,64−67^

### H/D Exchange Reaction Calculations

We computed single-point energies and corresponding zero-point energies from harmonic frequency calculations for H_2_, D_2_, HD, and the eight possible isotopomers/isotopologues of XOX·X^–^, where X is hydrogen or deuterium. The electronic structure was calculated with coupled cluster theory and explicit correlation^68−70^ using an appropriate triple-ζ basis set with diffuse and polarization functions,^71−73^ and the necessary auxiliary basis sets for explicit correlation.^74−78^ We refer to this setup as CCSD(T)-F12/aug-cc-pVTZ-F12. Based on the absolute energies, we calculate the reaction energies for hydrogen–deuterium exchange reactions from one XOX·X^–^ isotopomer with H_2_ and D_2_, each resulting in HD and another isotopomer of XOX·X^–^. All possible combinations are listed in Figure 2.

### Vibrational Frequency Calculations

We performed harmonic and anharmonic frequency calculations for spectral interpretation of all deuterated isotopomers and isotopologues of XOX·X^–^ and OX^–^·X_2_. For anharmonic calculations, we predominantly used vibrational perturbation theory of second order (VPT2) using a quartic force field (QFF) as a potential energy surface (PES). Additionally, we investigated vibrational self-consistent field and configuration interaction (VSCF/VCI) calculations for the HOH·H^–^ species, using a *n*-mode expansion of the PES with up to 4-mode couplings. All calculations rely on electronic structure calculations at the CCSD(T)-F12/aug-cc-pVTZ-F12 level of theory.

## Data Availability

The data that support the findings of this study are openly available at https://doi.org/10.5281/zenodo.10781113.

## Supporting Information Available

Calculated structural parameters, harmonic and VPT anharmonic frequencies for X_2_O ·X^–^ and XO^–^ ·X_2_ (X = H,D) up to 5000 cm^–1^. Single-point energies and harmonic zero-point energies for the H/D exchange reaction in X_2_O ·X^–^. VPT2 anharmonic zero-point energies for validation of the H/D exchange reaction energies. Convergence tests for anharmonic VSCF/VCI frequencies of H_2_O·H^–^ using a 4-mode PES (PDF)
