# Supplementary material for: Zero-Point-Energy Driven Isotopic Exchange of the [H3O]− anion Probed by Mid-Infrared Action Spectroscopy
Source: J Am Chem Soc. 2024 Jul 25;146(31):21634–41. doi: 10.1021/jacs.4c05543 (PMC11311240; doi:10.1021/jacs.4c05543)
Supplement: Supplementary file 1 — ja4c05543_si_001.pdf [file ja4c05543_si_001.pdf]

Supporting Information: Zero-point-energy driven isotopic exchange of the $[H_3O]^-$ anion probed by mid-Infrared action spectroscopy

Dennis F. Dinu,[†,‡] MilanÊnčák,[¶] Sven Thorwirth,[§] Klaus R. Liedl,[‡] Sandra Brünken,[‖] Stephan Schlemmer,[§] and Pavol Jusko[*,⊥]

†*Institute of Materials Chemistry, TU Wien, Getreidemarkt 9/165, 1060 Vienna, Austria*

‡*Department of General, Inorganic and Theoretical Chemistry, Universität Innsbruck, Innrain 80/82, 6020 Innsbruck, Austria*

¶*Institut für Ionenphysik und Angewandte Physik, Universität Innsbruck, Technikerstrasse 25, 6020 Innsbruck, Austria*

§*I. Physikalisches Institut, Universität zu Köln, Zülpicher Str. 77, 50937 Köln, Germany*

‖*Radboud University, FELIX Laboratory, Institute for Molecules and Materials, Toernooiveld 7, 6525 ED Nijmegen, The Netherlands*

⊥*Max Planck Institute for Extraterrestrial Physics, Giessenbachstrasse 1, 85748 Garching, Germany*

E-mail: pjusko@mpe.mpg.de

Contents

S1 Reaction pathway SI-3

S1.1 Structural parameters, zero point energies SI-3

S1.2 Deuterium-Hydrogen exchange reaction energies SI-5

S2 Harmonic approximation & Vibrational perturbation theory (VPT2) SI-8

S2.1 Normal modes of $HOH \cdot H^-$ in C_s symmetry SI-8

S2.2 Harmonic frequencies for different isotopomers SI-9

S2.3 VPT2 anharmonic frequencies for different isotopomers SI-9

S2.4 $XOX \cdot X^-$ Harmonic & VPT2 frequencies < 5000 cm^{-1} SI-10

S2.5 Normal modes of $HO^- \cdot H_2$ in C_s symmetry SI-17

S2.6 $XO^- \cdot XX$ Harmonic & VPT2 frequencies < 5000 cm^{-1} SI-18

S3 $H_2O \cdot H^-$: n-mode PES, VSCF and VCI SI-25

S3.1 Normal mode q_5 leads to troublesome PES SI-25

S3.2 Convergence tests for a multi-mode PES including mode q_5 . . . . . SI-26

S3.2.1 Removing parts in the polynomial multi-mode PES representation SI-26

S3.2.2 Limiting the expansion of the VCI configuration space SI-27

S3.3 Harmonic & VSCF frequencies up to 5000 cm^{-1} SI-33

S4 Appendix SI-40

S1 Reaction pathway

S1.1 Structural parameters, zero point energies

For the $[H_3O]^-$ anion, two energy minima exist along the reaction pathway

$$OX^- + X_2 \longleftrightarrow OX^- \cdot X_2 \longleftrightarrow X_2O \cdot X^- \longleftrightarrow X_2O + X^- \tag{S1}$$

where X=H,D. From this reaction pathway, we consider the two energy minima $XO^- \cdot X_2$ and $XOX \cdot X^-$ for further investigation. The structural parameters within the Born-Oppenheimer approximation are shown in Table S1.1. These are obtained from geometry optimization, where single point energies (SPE) are calculated at CCSD(T)-F12/AVTZ-F12 level of theory. For the various isotopic species, we compute the zero point energies (ZPE) within the harmonic approximation at CCSD(T)-F12/AVTZ-F12 level of theory using MOLPRO [1]. For the $XOX \cdot X^-$ species, we additionally compute ZPEs at MP2/AVTZ level of theory both in the harmonic approximation and by the VPT2 aprroach from Barone et al.[2] using GAUSSIAN [3]. All energies are listed in Table S1.2.

Table S1.1: Structural parameters (Born-Oppenheimer) for the two energy minima at CCSD(T)-F12/AVTZ-F12 level of theory. Distances (B1, B2, B3) in Å, angles (A1, A2) in degree.

	$XOX \cdot X^-$	$XO^- \cdot X_2$
	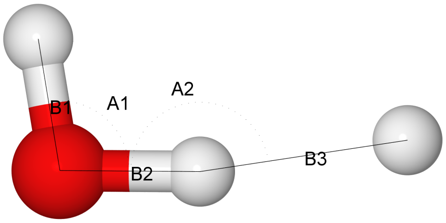	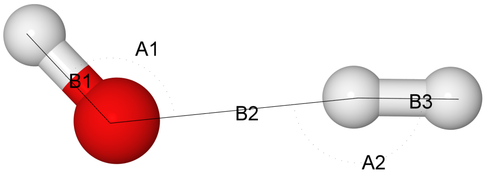
B1	0.9600	0.9644
B2	1.0134	1.9247
A1	100.04	128.47
B3	1.5151	0.7744
A2	170.46	186.32

[1] https://www.molpro.net/, https://doi.org/10.1063/5.0005081
[2] https://doi.org/10.1063/1.1824881
[3] https://gaussian.com/, Gaussian 16, Revision C.01, M. J. Frisch *et al.*, Gaussian, Inc., Wallingford CT, 2016.

Table S1.2: Calculated single point energies (SPE) and zero point energies (ZPE) in Hartree.

	Molpro CCSD(T)-F12/AVTZ-F12			Gaussian MP2/AVTZ				
Species	SPE	ZPE	SPE+ZPE	SPE	ZPE	SPE+ZPE	ZPE(VPT2)	SPE+ZPE
OD^-	-75.74168448	0.00621146	-75.73547302					
OH^-		0.00853226	-75.73315222					
DD	-1.17479285	0.00709311	-1.16769974	-1.16502310	0.007279793	-1.15774331	0.007233755	-1.15778934
DH		0.00868472	-1.16610813		0.008913606	-1.15610949	0.008844396	-1.15617870
HD		0.00868472	-1.16610813		0.008913606	-1.15610949	0.008844396	-1.15617870
HH		0.01002732	-1.16476553		0.010291227	-1.15473187	0.010198714	-1.15482439
OD^- + DD			-76.90317276					
OD^- + DH			-76.90158115					
OD^- + HD			-76.90158115					
OD^- + HH			-76.90023855					
OH^- + DD			-76.90085196					
OH^- + DH			-76.89926035					
OH^- + HD			-76.89926035					
OH^- + HH			-76.89791775					
$DO^- \cdot DD$	-76.92518173	0.01583449	-76.90934724					
$DO^- \cdot DH$		0.01777232	-76.90740941					
$DO^- \cdot HD$		0.0179844	-76.90719733					
$HO^- \cdot DD$		0.01824175	-76.90693998					
$DO^- \cdot HH$		0.01967349	-76.90550824					
$HO^- \cdot DH$		0.02017782	-76.90500391					
$HO^- \cdot HD$		0.02038978	-76.90479195					
$HO^- \cdot HH$		0.0220767	-76.90310503					
$DOD \cdot D^-$	-76.92787125	0.01724585	-76.9106254	-76.8744311	0.016933218	-76.85749788	0.016475689	-76.85795541
$DOD \cdot H^-$		0.01785056	-76.91002069		0.017604841	-76.85682626	0.017181321	-76.85724978
$HOD \cdot D^-$		0.0201838	-76.90768745		0.019854457	-76.85457664	0.019298853	-76.85513225
$DOH \cdot D^-$		0.02030065	-76.9075706		0.019829482	-76.85460162	0.019015383	-76.85541572
$HOD \cdot H^-$		0.02078052	-76.90709073		0.020518316	-76.85391278	0.020000345	-76.85443076
$DOH \cdot H^-$		0.02086621	-76.90700504		0.020452167	-76.85397893	0.01968716	-76.85474394
$HOH \cdot D^-$		0.02320275	-76.9046685		0.022719472	-76.85171163	0.02180007	-76.85263103
$HOH \cdot H^-$		0.02375822	-76.90411303		0.023333323	-76.85109778	0.022465423	-76.85196568

S1.2 Deuterium-Hydrogen exchange reaction energies

From the SPEs and ZPEs (cf. Tab. S1.2), we calculate $XOX \cdot X^-$ exchange reaction energies (cf. Tab. S1.3, S1.5, S1.7 for DD to HD, cf. Tab. S1.4, S1.6, S1.8 for HH to HD). The absolute reaction energies using harmonic ZPEs from CCSD(T)-F12/AVTZ-F12 (cf. Tab. S1.3, S1.4) are larger than their MP2/AVTZ counterpart (cf. Tab. S1.5, S1.6). For the latter, anharmonic ZPEs decrease the reaction energies (cf. Tab. S1.7, S1.8). The reaction trend is consistent for all calculations.

Table S1.3: DD to HD exchange reaction at CCSD(T)-F12/AVTZ-F12 level of theory using harmonic ZPEs and the Molpro software.

DD	-1.16769974		HD	-1.16610813		Hartree	meV	kJ/mol
+ $DOD \cdot H^-$	-76.91002069	⟶	+ $DOD \cdot D^-$	-76.9106254	=	0.00098690	26.9	2.591
+ $HOD \cdot D^-$	-76.90768745	⟶	+ $DOD \cdot D^-$	-76.9106254	=	-0.00134634	-36.6	-3.535
+ $DOH \cdot D^-$	-76.90757060	⟶	+ $DOD \cdot D^-$	-76.9106254	=	-0.00146319	-39.8	-3.842
+ $HOD \cdot H^-$	-76.90709073	⟶	+ $HOD \cdot D^-$	-76.9076875	=	0.00099489	27.1	2.612
+ $HOD \cdot H^-$	-76.90709073	⟶	+ $DOD \cdot H^-$	-76.9100207	=	-0.00133835	-36.4	-3.514
+ $DOH \cdot H^-$	-76.90700504	⟶	+ $DOH \cdot D^-$	-76.9075706	=	0.00102605	27.9	2.694
+ $DOH \cdot H^-$	-76.90700504	⟶	+ $DOD \cdot H^-$	-76.9100207	=	-0.00142404	-38.8	-3.739
+ $HOH \cdot D^-$	-76.90466850	⟶	+ $HOD \cdot D^-$	-76.9076875	=	-0.00142734	-38.8	-3.747
+ $HOH \cdot D^-$	-76.90466850	⟶	+ $DOH \cdot D^-$	-76.9075706	=	-0.00131049	-35.7	-3.441
+ $HOH \cdot H^-$	-76.90411303	⟶	+ $HOD \cdot H^-$	-76.9070907	=	-0.00138609	-37.7	-3.639
+ $HOH \cdot H^-$	-76.90411303	⟶	+ $DOH \cdot H^-$	-76.9070050	=	-0.00130040	-35.4	-3.414
+ $HOH \cdot H^-$	-76.90411303	⟶	+ $HOH \cdot D^-$	-76.9046685	=	0.00103614	28.2	2.720

Table S1.4: HH to HD exchange reaction at CCSD(T)-F12/AVTZ-F12 level of theory using harmonic ZPEs and the Molpro software.

HH	-1.16476553		HD	-1.16610813		Hartree	meV	kJ/mol
$DOD \cdot D^-$	-76.91062540	⟶	+ $HOD \cdot D^-$	-76.9076875	=	0.00159535	43.4	4.189
$DOD \cdot D^-$	-76.91062540	⟶	+ $DOH \cdot D^-$	-76.9075706	=	0.00171220	46.6	4.495
$DOD \cdot D^-$	-76.91062540	⟶	+ $DOD \cdot H^-$	-76.9100207	=	-0.00073789	-20.1	-1.937
$HOD \cdot D^-$	-76.90768745	⟶	+ $HOD \cdot H^-$	-76.9070907	=	-0.00074588	-20.3	-1.958
$HOD \cdot D^-$	-76.90768745	⟶	+ $HOH \cdot D^-$	-76.9046685	=	0.00167635	45.6	4.401
$DOH \cdot D^-$	-76.90757060	⟶	+ $DOH \cdot H^-$	-76.9070050	=	-0.00077704	-21.1	-2.040
$DOH \cdot D^-$	-76.90757060	⟶	+ $HOH \cdot D^-$	-76.9046685	=	0.00155950	42.4	4.094
$DOD \cdot H^-$	-76.91002069	⟶	+ $HOD \cdot H^-$	-76.9070907	=	0.00158736	43.2	4.168
$DOD \cdot H^-$	-76.91002069	⟶	+ $DOH \cdot H^-$	-76.9070050	=	0.00167305	45.5	4.393
$HOD \cdot H^-$	-76.90709073	⟶	+ $HOH \cdot H^-$	-76.9041130	=	0.00163510	44.5	4.293
$DOH \cdot H^-$	-76.90700504	⟶	+ $HOH \cdot H^-$	-76.9041130	=	0.00154941	42.2	4.068
$HOH \cdot D^-$	-76.90466850	⟶	+ $HOH \cdot H^-$	-76.9041130	=	-0.00078713	-21.4	-2.067

Table S1.5: DD to HD exchange reaction at MP2/AVTZ level of theory using harmonic ZPEs and the GAUSSIAN software.

DD	-1.15774331		HD	-1.15610949		Hartree	meV	kJ/mol
+ $DOD\cdot H^-$	-76.85682626	⟶	+ $DOD\cdot D^-$	-76.8574979	=	0.000962190	26.2	2.526
+ $HOD\cdot D^-$	-76.85457664	⟶	+ $DOD\cdot D^-$	-76.8574979	=	-0.001287434	-35.0	-3.380
+ $DOH\cdot D^-$	-76.85460162	⟶	+ $DOD\cdot D^-$	-76.8574979	=	-0.001262459	-34.4	-3.315
+ $HOD\cdot H^-$	-76.85391278	⟶	+ $HOD\cdot D^-$	-76.8545766	=	0.000969959	26.4	2.547
+ $HOD\cdot H^-$	-76.85391278	⟶	+ $DOD\cdot H^-$	-76.8568263	=	-0.001279665	-34.8	-3.360
+ $DOH\cdot H^-$	-76.85397893	⟶	+ $DOH\cdot D^-$	-76.8546016	=	0.001011135	27.5	2.655
+ $DOH\cdot H^-$	-76.85397893	⟶	+ $DOD\cdot H^-$	-76.8568263	=	-0.001213514	-33.0	-3.186
+ $HOH\cdot D^-$	-76.85171163	⟶	+ $HOD\cdot D^-$	-76.8545766	=	-0.001231204	-33.5	-3.233
+ $HOH\cdot D^-$	-76.85171163	⟶	+ $DOH\cdot D^-$	-76.8546016	=	-0.001256189	-34.2	-3.298
+ $HOH\cdot H^-$	-76.85109778	⟶	+ $HOD\cdot H^-$	-76.8539128	=	-0.001181199	-32.1	-3.101
+ $HOH\cdot H^-$	-76.85109778	⟶	+ $DOH\cdot H^-$	-76.8539789	=	-0.001247340	-33.9	-3.275
+ $HOH\cdot H^-$	-76.85109778	⟶	+ $HOH\cdot D^-$	-76.8517116	=	0.001019964	27.8	2.678

Table S1.6: HH to HD exchange reaction at MP2/AVTZ level of theory using harmonic ZPEs and the GAUSSIAN software.

HH	-1.15473187		HD	-1.15610949		Hartree	meV	kJ/mol
$DOD\cdot D^-$	-76.85749788	⟶	+ $HOD\cdot D^-$	-76.8545766	=	0.00154362	42.0	4.053
$DOD\cdot D^-$	-76.85749788	⟶	+ $DOH\cdot D^-$	-76.8546016	=	0.00151864	41.3	3.987
$DOD\cdot D^-$	-76.85749788	⟶	+ $DOD\cdot H^-$	-76.8568263	=	-0.00070600	-19.2	-1.854
$HOD\cdot D^-$	-76.85457664	⟶	+ $HOD\cdot H^-$	-76.8539128	=	-0.00071376	-19.4	-1.874
$HOD\cdot D^-$	-76.85457664	⟶	+ $HOH\cdot D^-$	-76.8517116	=	0.00148739	40.5	3.905
$DOH\cdot D^-$	-76.85460162	⟶	+ $DOH\cdot H^-$	-76.8539789	=	-0.00075494	-20.5	-1.982
$DOH\cdot D^-$	-76.85460162	⟶	+ $HOH\cdot D^-$	-76.8517116	=	0.00151237	41.2	3.971
$DOD\cdot H^-$	-76.85682626	⟶	+ $HOD\cdot H^-$	-76.8539128	=	0.00153585	41.8	4.032
$DOD\cdot H^-$	-76.85682626	⟶	+ $DOH\cdot H^-$	-76.8539789	=	0.00146970	40.0	3.859
$HOD\cdot H^-$	-76.85391278	⟶	+ $HOH\cdot H^-$	-76.8510978	=	0.00143739	39.1	3.774
$DOH\cdot H^-$	-76.85397893	⟶	+ $HOH\cdot H^-$	-76.8510978	=	0.00150354	40.9	3.948
$HOH\cdot D^-$	-76.85171163	⟶	+ $HOH\cdot H^-$	-76.8510978	=	-0.00076377	-20.8	-2.005

Table S1.7: DD to HD exchange reaction at MP2/AVTZ level of theory using anharmonic VPT2 ZPEs and the GAUSSIAN software.

DD	-1.15778934		HD	-1.15617870		Hartree	meV	kJ/mol
+ $DOD \cdot H^-$	-76.85724978	⟶	+ $DOD \cdot D^-$	-76.8579554	=	0.00090501	24.6	2.376
+ $HOD \cdot D^-$	-76.85513225	⟶	+ $DOD \cdot D^-$	-76.8579554	=	-0.00121252	-33.0	-3.183
+ $DOH \cdot D^-$	-76.85541572	⟶	+ $DOD \cdot D^-$	-76.8579554	=	-0.00092905	-25.3	-2.439
+ $HOD \cdot H^-$	-76.85443076	⟶	+ $HOD \cdot D^-$	-76.8551322	=	0.00090915	24.7	2.387
+ $HOD \cdot H^-$	-76.85443076	⟶	+ $DOD \cdot H^-$	-76.8572498	=	-0.00120838	-32.9	-3.173
+ $DOH \cdot H^-$	-76.85474394	⟶	+ $DOH \cdot D^-$	-76.8554157	=	0.00093886	25.5	2.465
+ $DOH \cdot H^-$	-76.85474394	⟶	+ $DOD \cdot H^-$	-76.8572498	=	-0.00089520	-24.4	-2.350
+ $HOH \cdot D^-$	-76.85263103	⟶	+ $HOD \cdot D^-$	-76.8551322	=	-0.00089058	-24.2	-2.338
+ $HOH \cdot D^-$	-76.85263103	⟶	+ $DOH \cdot D^-$	-76.8554157	=	-0.00117405	-31.9	-3.082
+ $HOH \cdot H^-$	-76.85196568	⟶	+ $HOD \cdot H^-$	-76.8544308	=	-0.00085444	-23.3	-2.243
+ $HOH \cdot H^-$	-76.85196568	⟶	+ $DOH \cdot H^-$	-76.8547439	=	-0.00116762	-31.8	-3.066
+ $HOH \cdot H^-$	-76.85196568	⟶	+ $HOH \cdot D^-$	-76.8526310	=	0.00094529	25.7	2.482

Table S1.8: HH to HD exchange reaction at MP2/AVTZ level of theory using anharmonic VPT2 ZPEs and the GAUSSIAN software.

HH	-1.15482439		HD	-1.15617870		Hartree	meV	kJ/mol
$DOD \cdot D^-$	-76.85795541	⟶	+ $HOD \cdot D^-$	-76.8551322	=	0.00146884	40.0	3.856
$DOD \cdot D^-$	-76.85795541	⟶	+ $DOH \cdot D^-$	-76.8554157	=	0.00118538	32.3	3.112
$DOD \cdot D^-$	-76.85795541	⟶	+ $DOD \cdot H^-$	-76.8572498	=	-0.00064869	-17.7	-1.703
$HOD \cdot D^-$	-76.85513225	⟶	+ $HOD \cdot H^-$	-76.8544308	=	-0.00065283	-17.8	-1.714
$HOD \cdot D^-$	-76.85513225	⟶	+ $HOH \cdot D^-$	-76.8526310	=	0.00114690	31.2	3.011
$DOH \cdot D^-$	-76.85541572	⟶	+ $DOH \cdot H^-$	-76.8547439	=	-0.00068254	-18.6	-1.792
$DOH \cdot D^-$	-76.85541572	⟶	+ $HOH \cdot D^-$	-76.8526310	=	0.00143037	38.9	3.755
$DOD \cdot H^-$	-76.85724978	⟶	+ $HOD \cdot H^-$	-76.8544308	=	0.00146471	39.9	3.846
$DOD \cdot H^-$	-76.85724978	⟶	+ $DOH \cdot H^-$	-76.8547439	=	0.00115152	31.3	3.023
$HOD \cdot H^-$	-76.85443076	⟶	+ $HOH \cdot H^-$	-76.8519657	=	0.00111076	30.2	2.916
$DOH \cdot H^-$	-76.85474394	⟶	+ $HOH \cdot H^-$	-76.8519657	=	0.00142395	38.7	3.739
$HOH \cdot D^-$	-76.85263103	⟶	+ $HOH \cdot H^-$	-76.8519657	=	-0.00068897	-18.7	-1.809

S2 Harmonic approximation & Vibrational perturbation theory (VPT2)

S2.1 Normal modes of $HOH \cdot H^-$ in C_s symmetry

When calculating the harmonic frequencies of the $HOH \cdot H^-$ complex in its planar conformation of C_s symmetry, we obtain the 6 normal modes, as shown in Table S2.1. The normal modes can be distinguished into two categories: intramolecular and intermolecular. Note that as the complex is composed of a molecule and an atom, the wording "intermolecular" is not valid here and is merely for communication purposes. The modes q_4, q_5, q_6 with frequencies above 1600 cm^{-1} are "localized" on the water sub-unit, and we may denote them as intramolecular. The normal modes q_1, q_2, q_3 with lower frequencies are intermolecular.

Table S2.1: Normal modes of $HOH \cdot H^-$ at CCSD(T)-F12/AVTZ-F12 level of theory.

q_1: H_2O "rotation"<br>495 cm^{-1}<br>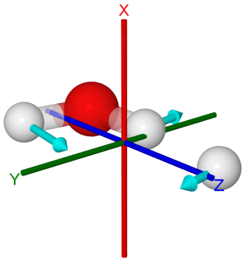	q_2: $HOH \cdot H^-$ "stretch"<br>639 cm^{-1}<br>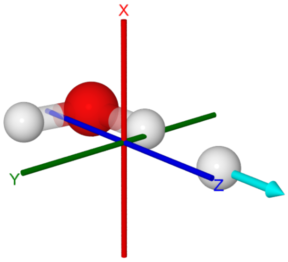	q_3: $HOH \cdot H^-$ "bending"<br>944 cm^{-1}<br>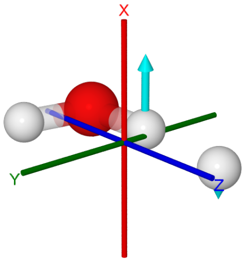
q_4: H_2O "bending"<br>1680 cm^{-1}<br>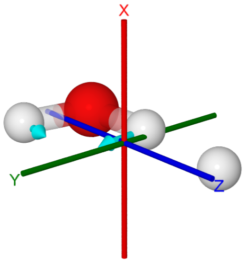	q_5: $OH \cdot H^-$ "stretching"<br>2821 cm^{-1}<br>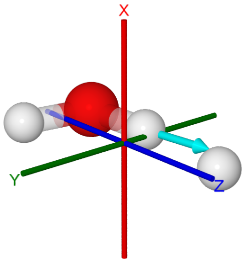	q_6: OH "stretching"<br>3581cm^{-1}<br>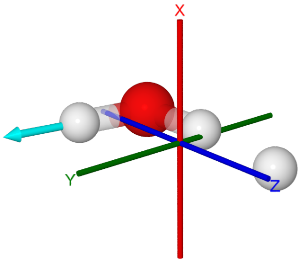

S2.2 Harmonic frequencies for different isotopomers

Table S2.2: Harmonic frequencies of the intermolecular modes calculated at CCSD(T)-F12/AVTZ-F12, in cm^{-1}.

	$q_1(A')$	$q_2(A')$	$q_3(A')$	$2q_1(A')$	$2q_2(A')$	$q_1+q_2(A')$
$HOH \cdot H^-$	492.5	641.7	943.1	985.0	1283.3	1134.1
$HOD \cdot H^-$	445.5	646.5	720.2	891.0	1273.0	1082.0
$DOH \cdot H^-$	410.3	637.2	941.2	820.6	1274.4	1047.5
$DOD \cdot H^-$	388.7	631.2	717.6	777.5	1262.3	1014.9
$HOH \cdot D^-$	449.1	482.8	911.7	898.3	965.7	932.0
$HOD \cdot D^-$	406.1	468.5	678.4	812.3	937.1	874.7
$DOH \cdot D^-$	380.3	463.3	909.7	760.6	926.5	843.6
$DOD \cdot D^-$	352.9	459.7	675.7	705.7	919.4	812.6
LIR: $21m/z$ | $20m/z$	571 | 584			1068 | 1100		

S2.3 VPT2 anharmonic frequencies for different isotopomers

As a first anharmonic correction, we calculate the PES as quartic force field (QFF) for subsequent vibrational perturbation theory (VPT2) calculations. Note that these calculations are limited in their comparability to the experiment, as they base on a rather local PES, using a Taylor series expansion. As shown in Section S3, the shape of the PES is actually dominated by a double minimum.

Table S2.3: Anharmonic VPT2 frequencies of the intermolecular modes calculated from a quartic force field at CCSD(T)-F12/AVTZ-F12, in cm^{-1}.

	$q_1(A')$	$q_2(A')$	$q_3(A')$	$2q_1(A')$	$2q_2(A')$	$q_1+q_2(A')$
$HOH \cdot H^-$	469.1	602.9	938.8	883.2	1156.9	1045.0
$HOD \cdot H^-$	431.8	590.1	719.3	818.5	1123.5	996.3
$DOH \cdot H^-$	394.0	590.4	925.5	756.2	1128.1	962.5
$DOD \cdot H^-$	371.7	582.1	706.9	711.7	1131.1	924.1
$HOH \cdot D^-$	424.5	486.6	915.6	820.2	950.8	880.6
$HOD \cdot D^-$	393.8	458.4	685.6	754.6	895.6	829.1
$DOH \cdot D^-$	365.5	445.3	902.3	699.5	866.0	798.0
$DOD \cdot D^-$	343.0	438.5	673.6	658.6	851.9	768.4
LIR: $21m/z$ | $20m/z$	571 | 584			1068 | 1100		

S2.4 XOX·X⁻ Harmonic & VPT2 frequencies < 5000 cm^{-1}

Tables S2.4 to S2.11 list calculated harmonic and VPT2 frequencies for the XOX·X⁻ species with $X = H, D$ in cm^{-1}. We computed vibrations with up to 2 excited quanta, i.e., fundamentals, first overtones, and combination bands.

Table S2.4: HOH·H⁻ at CCSD(T)-F12/AVTZ-F12.

Mode Irrep	Harmonic	VPT2	Intensity
6^1 A'	3851.86	3642.90	4.00
5^1 A'	2820.84	2340.46	68.34
4^1 A'	1678.44	1666.21	9.85
3^1 A"	943.13	938.78	172.45
2^1 A'	641.65	602.88	2099.96
1^1 A'	492.49	469.11	815.48
4^2 A'	3356.89	3116.30	0.16
3^2 A'	1886.25	1741.91	152.91
2^2 A'	1283.29	1156.90	62.56
1^2 A'	984.98	883.18	1.38
3^1+6^1 A"	4794.99	4581.63	0.07
2^1+6^1 A'	4493.51	4250.45	0.24
1^1+6^1 A'	4344.35	4096.21	0.16
3^1+5^1 A"	3763.96	3408.98	0.00
4^1+5^1 A'	4499.28	3332.53	3.87
2^1+5^1 A'	3462.48	2977.28	20.98
1^1+5^1 A'	3313.33	2843.22	91.61
3^1+4^1 A"	2621.57	2616.73	0.01
2^1+4^1 A'	2320.09	2135.60	18.99
1^1+4^1 A'	2170.94	1995.43	192.42
2^1+3^1 A"	1584.77	1500.96	0.01
1^1+3^1 A"	1435.62	1384.73	0.22
1^1+2^1 A'	1134.14	1044.98	52.90

Table S2.5: HOH·D⁻ at CCSD(T)-F12/AVTZ-F12.

Mode Irrep	Harmonic	VPT2	Intensity
6^1 A'	3851.85	3641.56	6.04
5^1 A'	2816.10	2272.25	161.53
4^1 A'	1672.77	1543.18	629.82

(Table continues on next page)

Continuation of Table S2.5

Mode	Harmonic	VPT2	Intensity
3^1 A"	911.69	915.58	80.06
2^1 A'	482.83	486.59	1431.18
1^1 A'	449.12	424.52	5252.38
4^2 A'	3345.54	3051.41	0.71
3^2 A'	1823.39	1675.01	107.45
2^2 A'	965.66	950.83	0.23
1^2 A'	898.25	820.22	3.00
$3^1 + 6^1$ A"	4763.54	4557.33	0.07
$2^1 + 6^1$ A'	4334.67	4163.86	0.02
$1^1 + 6^1$ A'	4300.97	4092.10	0.62
$3^1 + 5^1$ A"	3727.80	3338.39	0.01
$4^1 + 5^1$ A'	4488.87	3279.43	3.27
$2^1 + 5^1$ A'	3298.93	2840.31	7.24
$1^1 + 5^1$ A'	3265.23	2715.46	162.52
$3^1 + 4^1$ A"	2584.46	2576.85	0.03
$2^1 + 4^1$ A'	2155.60	2000.95	60.30
$1^1 + 4^1$ A'	2121.89	1941.56	157.53
$2^1 + 3^1$ A"	1394.52	1379.75	0.02
$1^1 + 3^1$ A"	1360.82	1316.55	0.08
$1^1 + 2^1$ A'	931.95	880.58	43.94

Table S2.6: $HOD \cdot H^-$ at CCSD(T)-F12/AVTZ-F12.

Mode Irrep	Harmonic	VPT2	Intensity
6^1 A'	3850.51	3646.78	5.05
5^1 A'	2065.84	1863.23	34.43
4^1 A'	1402.78	1330.95	69.61
3^1 A"	720.19	719.32	241.22
2^1 A'	636.47	590.13	1787.33
1^1 A'	445.50	431.80	433.32
5^2 A'	4131.68	2866.24	8.95
4^2 A'	2805.57	2609.25	4.99
3^2 A'	1440.39	1364.88	83.93
2^2 A'	1272.95	1123.53	151.81
1^2 A'	891.00	818.53	2.13
$3^1 + 6^1$ A"	4570.70	4366.32	0.07
$2^1 + 6^1$ A'	4486.98	4240.41	0.28

(Table continues on next page)

Continuation of Table S2.6

Mode	Harmonic	VPT2	Intensity
$1^1 + 6^1$ A'	4296.00	4076.95	0.47
$4^1 + 5^1$ A'	3468.63	3162.93	1.55
$3^1 + 5^1$ A"	2786.04	2659.89	0.00
$2^1 + 5^1$ A'	2702.31	2495.19	5.92
$1^1 + 5^1$ A'	2511.34	2347.56	34.75
$3^1 + 4^1$ A"	2122.98	2072.81	0.00
$2^1 + 4^1$ A'	2039.26	1934.13	2.62
$1^1 + 4^1$ A'	1848.28	1741.00	205.42
$2^1 + 3^1$ A"	1356.67	1277.69	0.00
$1^1 + 3^1$ A"	1165.69	1147.47	0.19
$1^1 + 2^1$ A'	1081.97	996.31	23.76

Table S2.7: $HOD \cdot D^-$ at CCSD(T)-F12/AVTZ-F12.

Mode Irrep	Harmonic	VPT2	Intensity
6^1 A'	3850.51	3646.04	5.94
5^1 A'	2058.54	1652.83	325.58
4^1 A'	1397.26	1379.02	84.96
3^1 A"	678.42	685.57	104.84
2^1 A'	468.53	458.36	904.83
1^1 A'	406.13	393.83	2342.54
5^2 A'	4117.08	2739.21	27.26
4^2 A'	2794.53	2697.69	0.90
3^2 A'	1356.84	1287.30	274.38
2^2 A'	937.06	895.58	7.87
1^2 A'	812.26	754.64	1.20
$3^1 + 6^1$ A"	4528.93	4331.64	0.08
$2^1 + 6^1$ A'	4319.04	4107.42	0.01
$1^1 + 6^1$ A'	4256.64	4039.26	0.77
$4^1 + 5^1$ A'	3455.81	2941.14	0.33
$3^1 + 5^1$ A"	2736.96	2568.37	0.01
$2^1 + 5^1$ A'	2527.07	2333.27	1.55
$1^1 + 5^1$ A'	2464.67	2242.90	20.50
$3^1 + 4^1$ A"	2075.69	2059.43	0.01
$2^1 + 4^1$ A'	1865.79	1847.44	9.99
$1^1 + 4^1$ A'	1803.40	1777.64	25.99
$2^1 + 3^1$ A"	1146.95	1127.36	0.01
$1^1 + 3^1$ A"	1084.55	1076.06	0.06

(Table continues on next page)

Continuation of Table S2.7

Mode	Harmonic	VPT2	Intensity
$1^1 + 2^1$ A'	874.66	829.07	43.99

Table S2.8: $DOH \cdot H^-$ at CCSD(T)-F12/AVTZ-F12.

Mode Irrep	Harmonic	VPT2	Intensity
5^1 A'	2776.26	2624.74	1076.82
6^1 A'	2844.66	2240.23	774.80
4^1 A'	1549.13	1483.34	19.03
3^1 A"	941.18	925.45	161.63
2^1 A'	637.22	590.35	1978.44
1^1 A'	410.31	393.99	240.19
4^2 A'	3098.26	3000.42	253.77
3^2 A'	1882.37	1769.79	82.31
2^2 A'	1274.44	1128.10	95.66
1^2 A'	820.61	756.24	0.71
$4^1 + 5^1$A'	4325.39	4181.94	1.07
$4^1 + 6^1$A'	4393.78	3714.43	3.81
$3^1 + 5^1$A"	3717.44	3562.44	0.03
$3^1 + 6^1$A"	3785.84	3327.38	0.00
$2^1 + 5^1$A'	3413.48	3195.11	50.06
$1^1 + 5^1$A'	3186.56	2992.73	3.72
$2^1 + 6^1$A'	3481.88	2877.15	370.52
$1^1 + 6^1$A'	3254.96	2746.35	510.80
$3^1 + 4^1$A"	2490.31	2397.91	0.01
$2^1 + 4^1$A'	2186.35	2067.86	7.57
$1^1 + 4^1$A'	1959.43	1826.33	63.11
$2^1 + 3^1$A"	1578.41	1472.98	0.00
$1^1 + 3^1$A"	1351.49	1293.44	0.32
$1^1 + 2^1$A'	1047.53	962.52	10.72

Table S2.9: $DOH \cdot D^-$ at CCSD(T)-F12/AVTZ-F12.

Mode Irrep	Harmonic	VPT2	Intensity
5^1 A'	2774.35	2716.28	2093.60
6^1 A'	2841.85	2245.92	1458.69
4^1 A'	1541.73	1480.83	21.94

(Table continues on next page)

Continuation of Table S2.9

Mode	Harmonic	VPT2	Intensity
3^1 A"	909.68	902.26	72.78
2^1 A'	463.26	445.32	995.22
1^1 A'	380.31	365.47	211.04
4^2 A'	3083.47	2958.62	70.82
3^2 A'	1819.37	1730.57	99.67
2^2 A'	926.52	865.95	23.31
1^2 A'	760.62	699.44	1.05
$4^1 + 6^1$A'	4383.58	4176.21	1.04
$4^1 + 5^1$A'	4316.08	3641.91	4.10
$3^1 + 5^1$A"	3684.03	3508.35	0.02
$3^1 + 6^1$A"	3751.53	3245.45	0.01
$2^1 + 5^1$A'	3237.61	3221.87	1.80
$1^1 + 6^1$A'	3222.16	2891.68	170.46
$1^1 + 5^1$A'	3154.66	2671.25	395.14
$2^1 + 6^1$A'	3305.11	2558.70	1100.38
$3^1 + 4^1$A"	2451.42	2358.73	0.02
$2^1 + 4^1$A'	2005.00	1914.29	1.51
$1^1 + 4^1$A'	1922.04	1784.18	482.63
$2^1 + 3^1$A"	1372.95	1319.43	0.00
$1^1 + 3^1$A"	1290.00	1244.45	0.12
$1^1 + 2^1$A'	843.57	797.93	14.20

Table S2.10: DOD · H^- at CCSD(T)-F12/AVTZ-F12.

Mode Irrep	Harmonic	VPT2	Intensity
6^1 A'	2801.18	2687.59	1.68
5^1 A'	2062.84	1876.39	18.35
4^1 A'	1233.33	1065.83	69.60
3^1 A"	717.62	706.86	224.43
2^1 A'	631.16	582.13	1656.17
1^1 A'	388.74	371.69	317.05
5^2 A'	4125.69	2874.38	28.48
4^2 A'	2466.66	2399.88	3.71
3^2 A'	1435.25	1334.34	81.62
2^2 A'	1262.31	1131.05	32.74
1^2 A'	777.47	711.74	3.71
$5^1 + 6^1$ A'	4864.03	4491.47	0.11

(Table continues on next page)

Continuation of Table S2.10

Mode	Harmonic	VPT2	Intensity
$4^1 + 6^1$ A'	4034.51	3886.20	0.58
$3^1 + 6^1$ A"	3518.81	3398.20	0.03
$2^1 + 6^1$ A'	3432.34	3277.85	0.23
$1^1 + 6^1$ A'	3189.92	3060.24	0.13
$4^1 + 5^1$ A'	3296.17	3000.38	0.66
$3^1 + 5^1$ A"	2780.47	2597.96	0.00
$2^1 + 5^1$ A'	2694.00	2424.38	9.87
$1^1 + 5^1$ A'	2451.58	2229.40	22.79
$3^1 + 4^1$ A"	1950.95	1901.77	0.00
$2^1 + 4^1$ A'	1864.48	1790.91	5.75
$1^1 + 4^1$ A'	1622.07	1542.36	80.00
$2^1 + 3^1$ A"	1348.78	1257.27	0.01
$1^1 + 3^1$ A"	1106.36	1063.76	0.25
$1^1 + 2^1$ A'	1019.89	924.13	18.57

Table S2.11: $DOD \cdot D^-$ at CCSD(T)-F12/AVTZ-F12.

Mode Irrep	Harmonic	VPT2	Intensity
6^1 A'	2801.18	2685.53	5.91
5^1 A'	2055.50	1756.67	210.93
4^1 A'	1224.89	1181.28	127.33
3^1 A"	675.71	673.59	93.60
2^1 A'	459.70	438.45	882.20
1^1 A'	352.87	342.98	202.43
5^2 A'	4111.01	2731.17	21.52
4^2 A'	2449.79	2358.94	0.52
3^2 A'	1351.41	1290.66	70.44
2^2 A'	919.39	851.90	27.49
1^2 A'	705.74	658.57	0.64
$5^1 + 6^1$ A'	4856.69	4433.19	0.18
$4^1 + 6^1$ A'	4026.08	3846.25	0.55
$3^1 + 6^1$ A"	3476.89	3358.10	0.03
$2^1 + 6^1$ A'	3260.88	3125.04	0.20
$1^1 + 6^1$ A'	3154.05	3026.13	0.09
$4^1 + 5^1$ A'	3280.40	2937.00	0.85
$3^1 + 5^1$ A"	2731.21	2519.67	0.01
$2^1 + 5^1$ A'	2515.20	2226.85	19.49
$1^1 + 5^1$ A'	2408.38	2148.89	41.47

(Table continues on next page)

Continuation of Table S2.11

Mode	Harmonic	VPT2	Intensity
$3^1 + 4^1$ A”	1900.60	1902.39	0.01
$2^1 + 4^1$ A’	1684.59	1617.07	8.34
$1^1 + 4^1$ A’	1577.77	1503.94	107.94
$2^1 + 3^1$ A”	1135.40	1091.30	0.00
$1^1 + 3^1$ A”	1028.58	1005.60	0.09
$1^1 + 2^1$ A’	812.57	768.36	13.20

S2.5 Normal modes of $HO^- \cdot H_2$ in C_s symmetry

When calculating the harmonic frequencies of the $HO^- \cdot H_2$ complex in its planar conformation of C_s symmetry, we obtain the 6 normal modes, as shown in Table S2.12. The normal modes can be distinguished in two categories: intramolecular and intermolecular. The modes q_5, q_6 with frequencies above 3500 cm^{-1} are "localized" on either the OH^- or H_2 sub-unit, and we may denote them as intramolecular. The normal modes q_1, q_2 with frequencies below 400 cm^{-1} are intermolecular "stretches", and the normal modes q_3, q_4 are rotations of the H_2 sub-unit with respect to the OH^- sub-unit.

Table S2.12: Normal modes of $HO^- \cdot H_2$ at CCSD(T)-F12/AVTZ-F12 level of theory.

<table>
<tr><td>q_1: $HO^- \cdot H_2$ "bending" 120 cm^{-1}</td><td>q_2: $HO^- \cdot H_2$ "stretching" 351 cm^{-1}</td></tr>
<tr><td>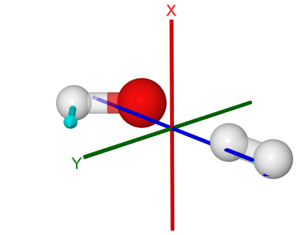
</td><td>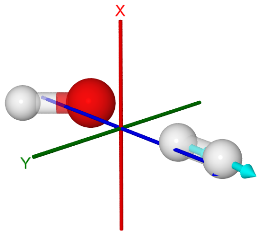
</td></tr>
<tr><td>q_3: H_2 "out of plane rotation" 802 cm^{-1}</td><td>q_4: H_2 "in plane rotation" 843 cm^{-1}</td></tr>
<tr><td>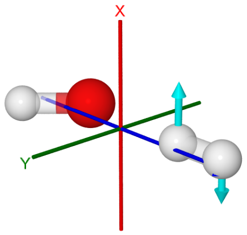
</td><td>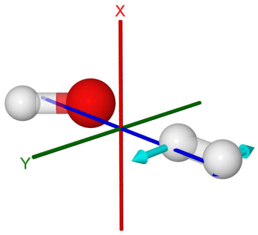
</td></tr>
<tr><td>q_5: OH^- "stretching" 3759 cm^{-1}</td><td>q_6: H_2 "stretching" 3816 cm^{-1}</td></tr>
<tr><td>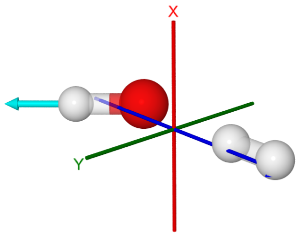
</td><td>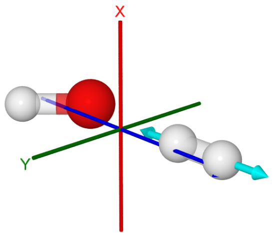
</td></tr>
</table>

S2.6 $XO^- \cdot XX$ Harmonic & VPT2 frequencies < 5000 cm^{-1}

Tables S2.13 to S2.20 list calculated harmonic and VPT2 frequencies for the $XO^- \cdot XX$ species with $X = H, D$ in cm^{-1}. We computed vibrations with up to 2 excited quanta, i.e., fundamentals, first overtones, and combination bands.

The VPT2 calculations for all isotopomers of the $XO^- \cdot XX$ system show one common problem: the frequency for the q_1 mode is "negative". In some cases, also the q_2 comes with a "negative" VPT2 frequency. It is well-known that large amplitude motions, usually with a harmonic frequency below 150 cm^{-1}, can lead to such negative anharmonic (over)corrections [4]. Especially studies on molecular clusters are prone to such problems, and the modes with "negative" VPT frequencies are then usually neglected in the analyis [5].

In the present work, we do not assign any of the $XO^- \cdot XX$ vibrations to our experimental spectrum. Nevertheless, we present our VPT2 calculations in Table S2.13 to Table S2.20 for the sake of completeness and to demonstrate the limitations of VPT2 for the $XO^- \cdot XX$ system.

Table S2.13: $HO^- \cdot HH$ at CCSD(T)-F12/AVTZ-F12.

Mode Irrep	Harmonic	VPT2	Intensity
5^1 A'	3758.49	3567.12	986.06
6^1 A'	3814.71	3345.55	47332.41
3^1 A"	808.10	624.70	0.44
4^1 A'	847.16	384.13	3857270.84
2^1 A'	350.77	283.79	37504.49
1^1 A'	158.97	-859.42	-8778.38
4^2 A'	1694.32	1103.99	0.00
3^2 A'	1616.20	828.92	108.13
2^2 A'	701.54	522.53	7.31
1^2 A'	317.94	-2739.21	-107.81
4^1+6^1 A'	4661.87	4374.04	2.11
3^1+6^1 A"	4622.81	4293.80	2.34
4^1+5^1 A'	4605.65	4218.21	0.16
3^1+5^1 A"	4566.59	4190.76	0.01
2^1+5^1 A'	4109.26	3840.30	1.10

(Table continues on next page)

[4] https://doi.org/10.1016/j.saa.2024.123969
[5] https://doi.org/10.1016/j.chemphys.2018.04.003

Continuation of Table S2.13

Mode	Harmonic	VPT2	Intensity
$2^1 + 6^1$ A'	4165.48	3656.54	211.17
$1^1 + 5^1$ A'	3917.46	2735.34	30.36
$1^1 + 6^1$ A'	3973.68	2512.28	437.86
$3^1 + 4^1$ A"	1655.26	1212.56	0.00
$2^1 + 4^1$ A'	1197.93	903.02	0.88
$2^1 + 3^1$ A"	1158.87	878.29	0.37
$1^1 + 4^1$ A'	1006.13	-236.57	-0.55
$1^1 + 3^1$ A"	967.07	-256.43	-0.20
$1^1 + 2^1$ A'	509.74	-578.40	-2.96

Table S2.14: $HO^- \cdot HD$ at CCSD(T)-F12/AVTZ-F12.

Mode Irrep	Harmonic	VPT2	Intensity
6^1 A'	3759.12	3572.40	821.11
5^1 A'	3271.52	2916.86	400.86
4^1 A'	776.26	616.51	3700812.62
3^1 A"	732.85	599.54	0.10
2^1 A'	297.85	-144.98	-8830.60
1^1 A'	159.51	-917.17	-22284.06
3^2 A'	1465.69	1103.93	0.10
4^2 A'	1552.51	839.44	97.92
2^2 A'	595.69	470.16	5.50
1^2 A'	319.02	-2908.19	-30.91
$4^1 + 6^1$ A'	4535.38	4193.04	0.05
$3^1 + 6^1$ A"	4491.97	4170.21	0.02
$4^1 + 5^1$ A'	4047.77	3875.99	0.80
$2^1 + 6^1$ A'	4056.97	3821.52	0.27
$3^1 + 5^1$ A"	4004.36	3797.55	0.73
$2^1 + 5^1$ A'	3569.36	3193.51	44.20
$1^1 + 6^1$ A'	3918.64	2687.89	6.14
$1^1 + 5^1$ A'	3431.03	2005.54	302.72
$3^1 + 4^1$ A"	1509.10	1166.10	0.00
$2^1 + 4^1$ A'	1074.10	845.66	0.53
$2^1 + 3^1$ A"	1030.69	825.31	0.06
$1^1 + 4^1$ A'	935.77	-319.82	-0.36
$1^1 + 3^1$ A"	892.36	-335.61	-0.06

(Table continues on next page)

Continuation of Table S2.14

Mode	Harmonic	VPT2	Intensity
$1^1 + 2^1$ A'	457.36	-668.58	-5.34

Table S2.15: $HO^- \cdot DH$ at CCSD(T)-F12/AVTZ-F12.

Mode Irrep	Harmonic	VPT2	Intensity
6^1 A'	3759.12	3572.19	884.68
5^1 A'	3338.46	3009.02	2754.36
4^1 A'	689.13	603.39	2998291.47
3^1 A"	664.30	553.49	0.98
2^1 A'	294.15	269.69	107760.51
1^1 A'	157.38	-861.93	-36719.81
4^2 A'	1378.27	1040.47	0.02
3^2 A'	1328.59	822.02	69.35
2^2 A'	588.29	461.80	5.74
1^2 A'	314.76	-2743.77	-40.05
$4^1 + 6^1$ A'	4448.25	4144.10	0.04
$3^1 + 6^1$ A"	4423.42	4124.24	0.02
$2^1 + 6^1$ A'	4053.27	3816.98	0.34
$4^1 + 5^1$ A'	4027.59	3804.83	2.68
$3^1 + 5^1$ A"	4002.75	3753.50	2.53
$2^1 + 5^1$ A'	3632.60	3273.53	31.94
$1^1 + 6^1$ A'	3916.50	2742.55	6.38
$1^1 + 5^1$ A'	3495.84	2153.20	176.89
$3^1 + 4^1$ A"	1353.43	1119.71	0.00
$2^1 + 4^1$ A'	983.28	796.54	1.00
$2^1 + 3^1$ A"	958.44	777.39	0.52
$1^1 + 4^1$ A'	846.51	-307.30	-1.07
$1^1 + 3^1$ A"	821.67	-324.27	-0.35
$1^1 + 2^1$ A'	451.52	-619.14	-4.71

Table S2.16: $HO^- \cdot DD$ at CCSD(T)-F12/AVTZ-F12.

Mode Irrep	Harmonic	VPT2	Intensity
6^1 A'	3759.08	3572.39	899.00

(Table continues on next page)

Continuation of Table S2.16

Mode	Harmonic	VPT2	Intensity
5^1 A'	2698.76	2480.27	8.34
3^1 A"	571.22	506.15	0.27
4^1 A'	600.44	462.85	928267.19
2^1 A'	264.35	230.89	27486.16
1^1 A'	156.90	-889.44	-77352.70
4^2 A'	1200.88	991.28	0.01
3^2 A'	1142.44	788.92	50.73
2^2 A'	528.71	436.73	4.82
1^2 A'	313.80	-2830.63	-23.32
$4^1 + 6^1$ A'	4359.51	4094.99	0.05
$3^1 + 6^1$ A"	4330.30	4077.14	0.04
$2^1 + 6^1$ A'	4023.43	3801.76	0.44
$4^1 + 5^1$ A'	3299.20	3187.25	0.80
$3^1 + 5^1$ A"	3269.98	3137.29	0.78
$2^1 + 5^1$ A'	2963.11	2730.39	14.49
$1^1 + 6^1$ A'	3915.98	2715.11	6.22
$1^1 + 5^1$ A'	2855.66	1593.30	88.02
$3^1 + 4^1$ A"	1171.66	1028.00	0.00
$2^1 + 4^1$ A'	864.79	735.21	0.50
$2^1 + 3^1$ A"	835.58	718.79	0.11
$1^1 + 4^1$ A'	757.34	-380.80	-0.81
$1^1 + 3^1$ A"	728.12	-395.15	-0.12
$1^1 + 2^1$ A'	421.25	-656.44	-6.79

Table S2.17: $DO^- \cdot HH$ at CCSD(T)-F12/AVTZ-F12.

Mode Irrep	Harmonic	VPT2	Intensity
6^1 A'	3813.77	3283.96	188501.07
5^1 A'	2736.72	2637.40	748.68
4^1 A'	846.50	615.59	229242932.37
3^1 A"	807.48	595.24	0.44
2^1 A'	349.56	255.41	483023.90
1^1 A'	102.20	-313.60	-765.29
3^2 A'	1614.95	977.67	0.19

(Table continues on next page)

Continuation of Table S2.17

Mode	Harmonic	VPT2	Intensity
4^2 A'	1693.01	760.32	98.79
2^2 A'	699.12	499.54	4.05
1^2 A'	204.41	-1031.10	-8.01
4^1+6^1 A'	4660.28	4346.95	2.12
3^1+6^1 A"	4621.25	4262.73	2.51
2^1+6^1 A'	4163.33	3569.07	64.87
4^1+5^1 A'	3583.22	3254.00	0.07
3^1+5^1 A"	3544.19	3232.29	0.00
1^1+6^1 A'	3915.98	2997.46	1368.75
2^1+5^1 A'	3086.28	2904.05	0.03
1^1+5^1 A'	2838.92	2335.85	2.72
3^1+4^1 A"	1653.98	1087.04	0.00
2^1+4^1 A'	1196.06	876.06	0.76
2^1+3^1 A"	1157.04	854.35	0.45
1^1+4^1 A'	948.71	283.68	490195.01
1^1+3^1 A"	909.68	254.98	0.50
1^1+2^1 A'	451.76	-36.39	-0.06

Table S2.18: $DO^- \cdot HD$ at CCSD(T)-F12/AVTZ-F12.

Mode Irrep	Harmonic	VPT2	Intensity
6^1 A'	3271.38	2870.83	38565.99
5^1 A'	2736.71	2637.98	924.53
4^1 A'	775.30	585.35	60618779.77
3^1 A"	731.89	568.45	0.22
2^1 A'	296.29	250.14	150499.61
1^1 A'	101.29	-382.75	-1485.33
3^2 A'	1463.78	977.43	0.57
4^2 A'	1550.60	769.75	89.16
2^2 A'	592.58	468.78	4.89
1^2 A'	202.58	-1237.80	-13.15
4^1+6^1 A'	4046.68	3852.60	0.72
3^1+6^1 A"	4003.27	3771.12	0.84
4^1+5^1 A'	3512.01	3224.39	0.21

(Table continues on next page)

Continuation of Table S2.18

Mode	Harmonic	VPT2	Intensity
3^1+5^1 A"	3468.60	3205.99	0.00
2^1+6^1 A'	3567.67	3131.68	49.59
2^1+5^1 A'	3033.00	2887.21	356.19
1^1+6^1 A'	3372.67	2502.41	961.26
1^1+5^1 A'	2838.00	2267.79	2.32
3^1+4^1 A"	1507.19	1039.59	0.00
2^1+4^1 A'	1071.59	814.20	0.49
2^1+3^1 A"	1028.18	797.05	0.10
1^1+4^1 A'	876.59	173.58	2037.72
1^1+3^1 A"	833.18	164.65	0.09
1^1+2^1 A'	397.58	-137.15	-0.43

Table S2.19: DO$-\cdot$DH at CCSD(T)-F12/AVTZ-F12.

Mode Irrep	Harmonic	VPT2	Intensity
6^1 A'	3338.33	2971.09	81159.16
5^1 A'	2736.71	2637.65	905.96
4^1 A'	688.17	545.24	56817911.17
3^1 A"	663.25	528.40	1.18
2^1 A'	292.21	244.32	199339.78
1^1 A'	101.43	-333.24	-1722.14
4^2 A'	1376.35	938.98	0.00
3^2 A'	1326.50	777.31	65.53
2^2 A'	584.41	457.32	4.98
1^2 A'	202.87	-1093.12	-11.61
4^1+6^1 A'	4026.51	3780.45	2.53
3^1+6^1 A"	4001.58	3727.79	2.75
2^1+6^1 A'	3630.54	3220.70	36.06
4^1+5^1 A'	3424.88	3183.45	0.35
3^1+5^1 A"	3399.96	3165.63	0.00
2^1+5^1 A'	3028.92	2880.80	0.09
1^1+6^1 A'	3439.77	2655.57	545.47
1^1+5^1 A'	2838.15	2316.62	2.47
3^1+4^1 A"	1351.42	1019.15	0.00
2^1+4^1 A'	980.38	772.15	0.98
2^1+3^1 A"	955.46	754.25	0.65

(Table continues on next page)

Continuation of Table S2.19

Mode	Harmonic	VPT2	Intensity
1^1+4^1 A'	789.61	188.37	2143.51
1^1+3^1 A"	764.68	175.85	0.43
1^1+2^1 A'	393.64	-93.36	-0.21

Table S2.20: DO−·DD at CCSD(T)-F12/AVTZ-F12.

Mode Irrep	Harmonic	VPT2	Intensity
6^1 A'	2736.80	2638.02	1997.91
5^1 A'	2698.45	2451.15	9528.86
4^1 A'	599.20	496.28	11833902.29
3^1 A"	569.98	481.80	0.59
2^1 A'	262.27	225.87	25485.18
1^1 A'	100.47	-379.70	-3951.98
3^2 A'	1139.96	895.04	0.05
4^2 A'	1198.40	748.76	48.00
2^2 A'	524.54	426.85	4.29
1^2 A'	200.93	-1231.45	-17.71
4^1+5^1 A'	3297.65	3163.89	0.81
4^1+6^1 A'	3336.00	3135.78	0.03
3^1+6^1 A"	3306.78	3120.07	0.01
3^1+5^1 A"	3268.43	3113.59	0.90
2^1+6^1 A'	2999.07	2862.62	0.12
2^1+5^1 A'	2960.72	2687.84	17.45
1^1+6^1 A'	2837.27	2270.63	1.08
1^1+5^1 A'	2798.92	2080.12	299.75
3^1+4^1 A"	1169.18	932.01	0.00
2^1+4^1 A'	861.47	707.49	0.48
2^1+3^1 A"	832.25	692.54	0.17
1^1+4^1 A'	699.66	97.00	0.29
1^1+3^1 A"	670.45	87.22	0.07
1^1+2^1 A'	362.73	-156.58	-0.62

S3 $H_2O \cdot H^-$: n-mode PES, VSCF and VCI

The anharmonic calculations from a quartic force field (QFF) and vibrational perturbation theory (VPT2) as shown in Section S2 are inherently limited, as they rely on a rather local description of the PES. We calculate a multi-mode PES as an alternative, where the potential energy is calculated along normal mode displacements. Although this is still a rather local description of the PES, it is considered to converge better than a Taylor series expansion. However, for the $HOH \cdot H^-$ system, the multi-mode PES representation based on rectilinear coordinates is expected to be problematic for the low frequency normal modes.

In the following, we elaborate on the convergence of anharmonic frequencies based on a multi-mode PES, using the vibrational self-consistent field (VSCF) and configuration interaction (VCI) approaches. These results demonstrate the difficulty of converging VSCF/VCI calculations for the multi-mode PES used here. These difficulties motivate us to rely on the VPT2 calculations instead of the VSCF/VCI calculations for interpreting the experimental data.

S3.1 Normal mode q_5 leads to troublesome PES

The multi-mode expansion with the default settings in XSURF generates the PES as shown in Table S4.1 in the Appendix. We want to highlight the 1D grid representation (cf. Figure S3.1). It demonstrates that for mode q_5, a double-well potential shape must be incorporated. This reflects the reaction:

$$HOH + H^- \longleftrightarrow HOH \cdot H^- \longleftrightarrow HO^- \cdot H_2 \longleftrightarrow HO^- + H_2 \qquad \text{(S2)}$$

Considering the present calculations, this particular mode causes troubles when fitting polynomials to the calculated multi-mode grid representation. It has to be expected that any VCI calculation based on such PES will be difficult to handle, thus, convergence test must be performed. Those are discussed in Section S3.2. Alternatively, the multi-mode PES could be expanded with the constraint of avoiding the double-well potential in mode q_5.

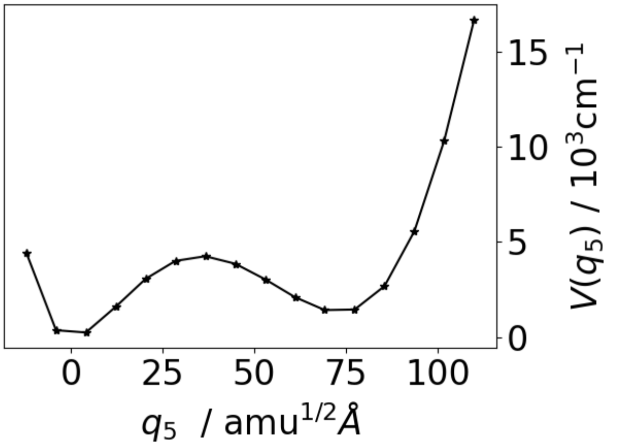

Figure S3.1: The XSURF algorithm generates a double-well potential for normal mode q_5 choosing relatively "harsh" parameters in scaling and shifting degree of displacement of the normal mode (sfac=1.850, shift= 6). The left minimum describes the equilibrium structure used for the PES expansion $HOH \cdot H^-$. The right minimum is most likely the second equilibrium structure $HO^- \cdot H_2$.

S3.2 Convergence tests for a multi-mode PES including mode q_5

S3.2.1 Removing parts in the polynomial multi-mode PES representation

In order to minimize the errors due to poor fits, we tested automatic deletion of troublesome potentials during the fit by using the `delauto` keyword. Table S3.1 shows three different polynomial fits and the results of subsequent VSCF calculations relying on these polynomial PES representation.

While the actual 1D polynomial representations (Table S3.1, POLY, blue) do not change significantly upon increasing the **delauto** threshold, the resulting ca-VSCF effective potentials (Table S3.1, VSCF, green) indicate significant changes in the PES upon increasing the **delauto** threshold. In the case of `delauto=1.d-5`, all effective cs-VSCF potentials show positive slopes at the edges. This is preferable for the subsequent VCI calculations, where configurations are generated by "excitations" from the VSCF reference. Still, normal mode q_5 is prone to introduce problematic configurations due to its double-well potential shape. Hence, further VCI convergence test are performed using these POLY settings in Section S3.2.2.

Table S3.1: 3-mode PES of (HOH)H- using CCSD(T)-F12/AVTZ-F12. The 1-mode potentials are shown as grid and polynomial representation. The 3-mode PES is shown via effective potentials from ca-VSCF.

delauto=off		delauto=1.d-10		delauto=1.d-5	
POLY	VSCF	POLY	VSCF	POLY	VSCF

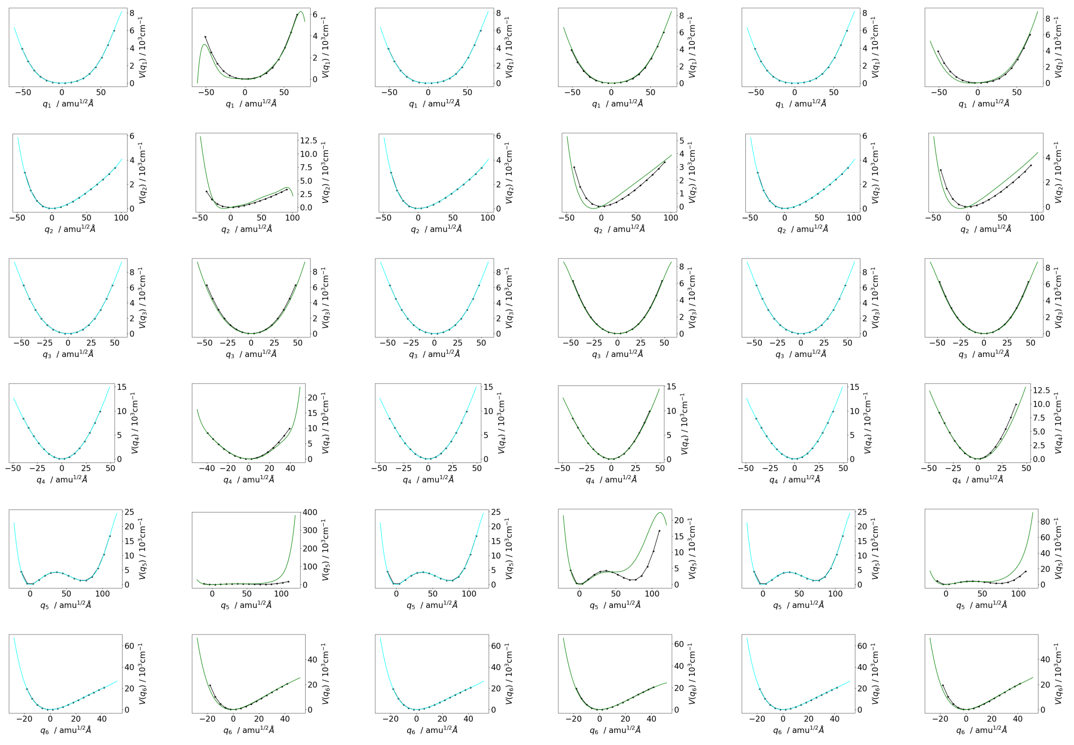

S3.2.2 Limiting the expansion of the VCI configuration space

Figure S3.2 depicts how we limit the configuration space in VCI using the `levex` directive. The configuration shown in the Figure is can be described by a vector $(1, 3, 4, 2, 2, 1)$, where the index i of the vector elements denote the normal mode q_i. In this particular configuration, all modes are in their *maximum* level of excitations defined by the user via the `levex` keyword. That means, there are multiple configurations with lower excitations, which are all considered in the VCI calculation. In the following, we discuss the influence of the `levex` directive, by limiting all possible modes separately.

The convergence test from various VCI calculations for vibrational transitions below 2000 cm^{-1} are depicted in Figure S3.3 for the fundamentals and in Figure S3.4 for the overtones. In this

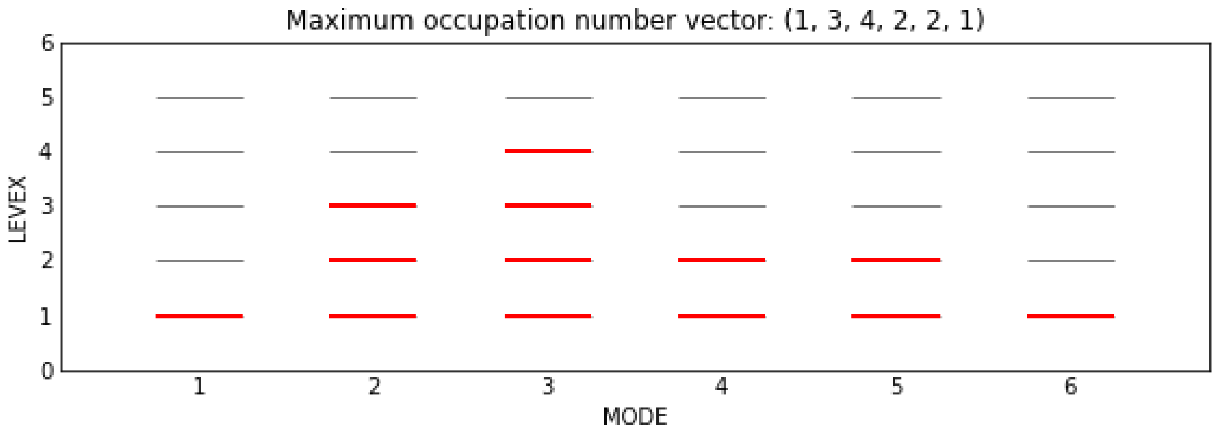

Figure S3.2: Exemplary depiction of maximum excitations allowed for a VCI calculation using the `levex` directive. Each mode is allowed to be excited to a certain amount: Mode q_1 to 1, mode q_2 to 4, mode q_3 to 4, etc. All together, the total maximum excitations are limited to 15.

benchmark, we investigate the change of the VCI transition frequency with the maximum level of excitation. Each calculated frequencies is associated with a combination of VCI configurations, where one configuration has the highest leading coefficient. If the relative leading coefficient is above 0.95, the assignment can be taken as reliable. In Figure S3.3 we use a color-scale to highlight "reliable" transition frequencies. Lower leading coefficients can indicate physically sound resonances **or** numerical inaccuracies in the calculation and, thus, such transition frequencies must be evaluated carefully.

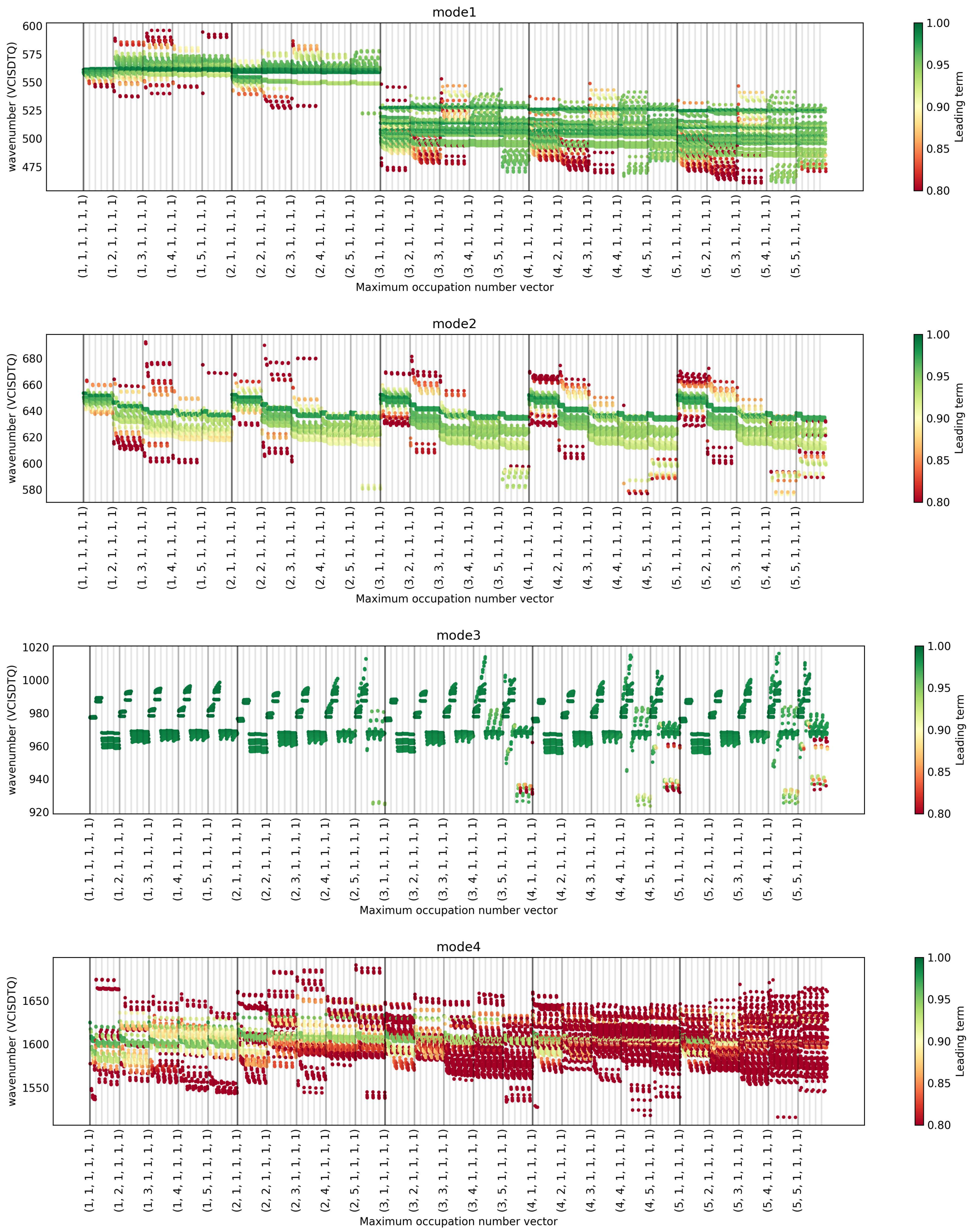

Figure S3.3: Benchmark for the VCI calculated fundamental transitions frequencies to $\nu_1(A')$, $\nu_2(A')$, $\nu_3(A'')$, and $\nu_4(A')$ with respect to the maximum level of excitation in generating the VCI configurations. Reliable assignments are green.

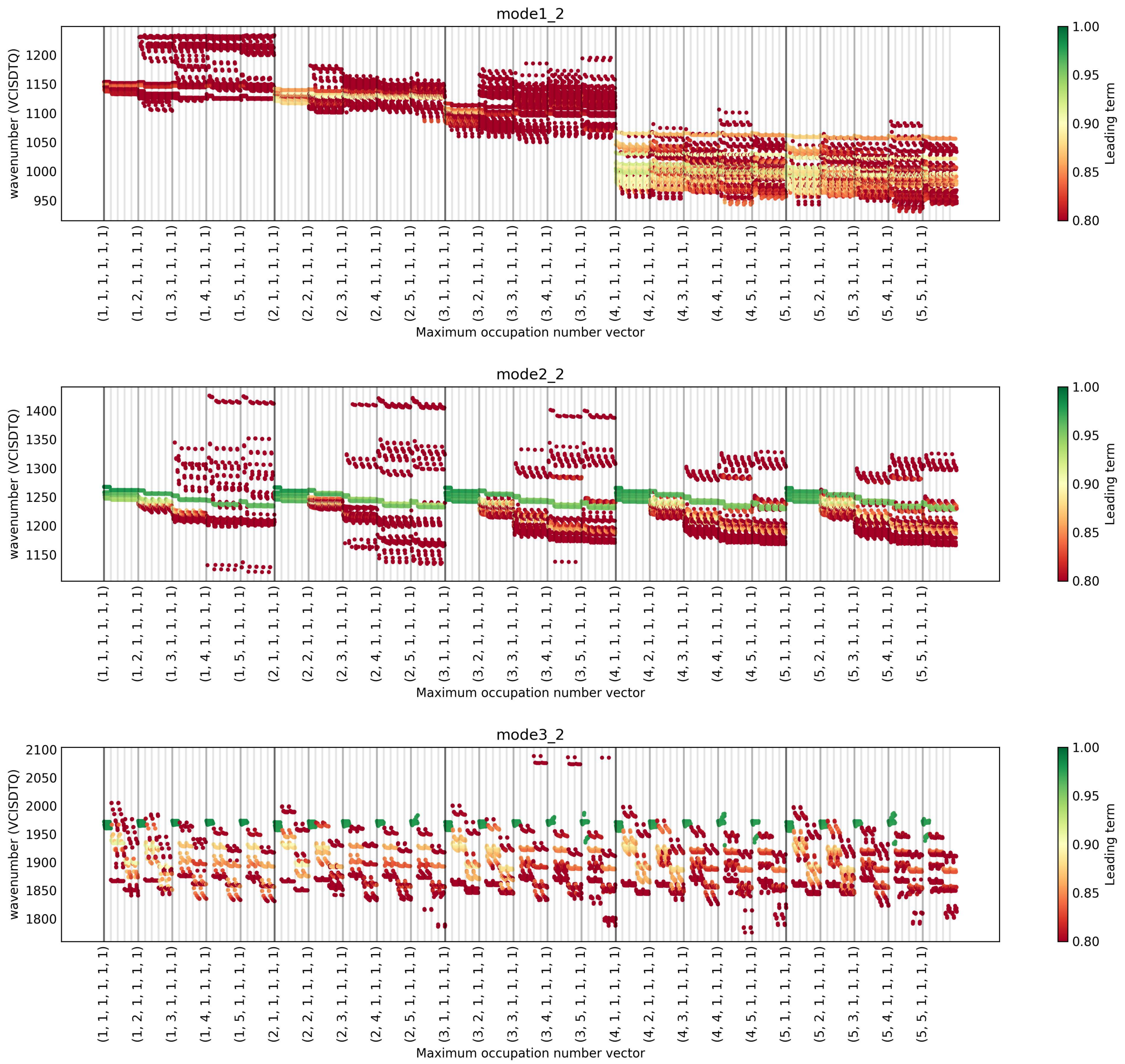

Figure S3.4: Benchmark for the VCI calculated overtone transitions frequencies to $2\nu_1(A')$, $2\nu_2(A')$, and $2\nu_3(A'')$ with respect to the maximum level of excitation in generating the VCI configurations. Reliable assignments are green.

In this first evaluation, it becomes already clear that increasing the level of excitations in the "higher" normal modes leads to divergence. From the discussion on the multi-mode PES in Section S3.2.1, we can expect that especially the mode q_5 causes troubles due to its double-well potential. Hence, it may be useful to simply prohibit excitations in this mode.

For the fundamental transition $\nu_1(A')$, figure S3.5 depicts the previous convergence tests with

the constraint of `levex=1` for one mode, while letting the other modes vary up to 5. In these plots, we denote the level of excitation alphabetically: For the mode q_1 with the letter J, for mode q_2 with the letter K, and so forth. Note the impact of limiting excitations in mode q_5 to 1, where all "unreliable" assignments vanish. Figure S3.6 shows the same evaluation for the overtone $\nu_1^2(A')$. Again, limiting excitations in mode q_5 to 1 yields the most "reliable" results. However, here it shows to be rather important to allow higher excitations in all the other modes. In general, it appears that there is a resonance for the fundamental and its overtone, however, this has to be further investigated using other approaches than the one used here.

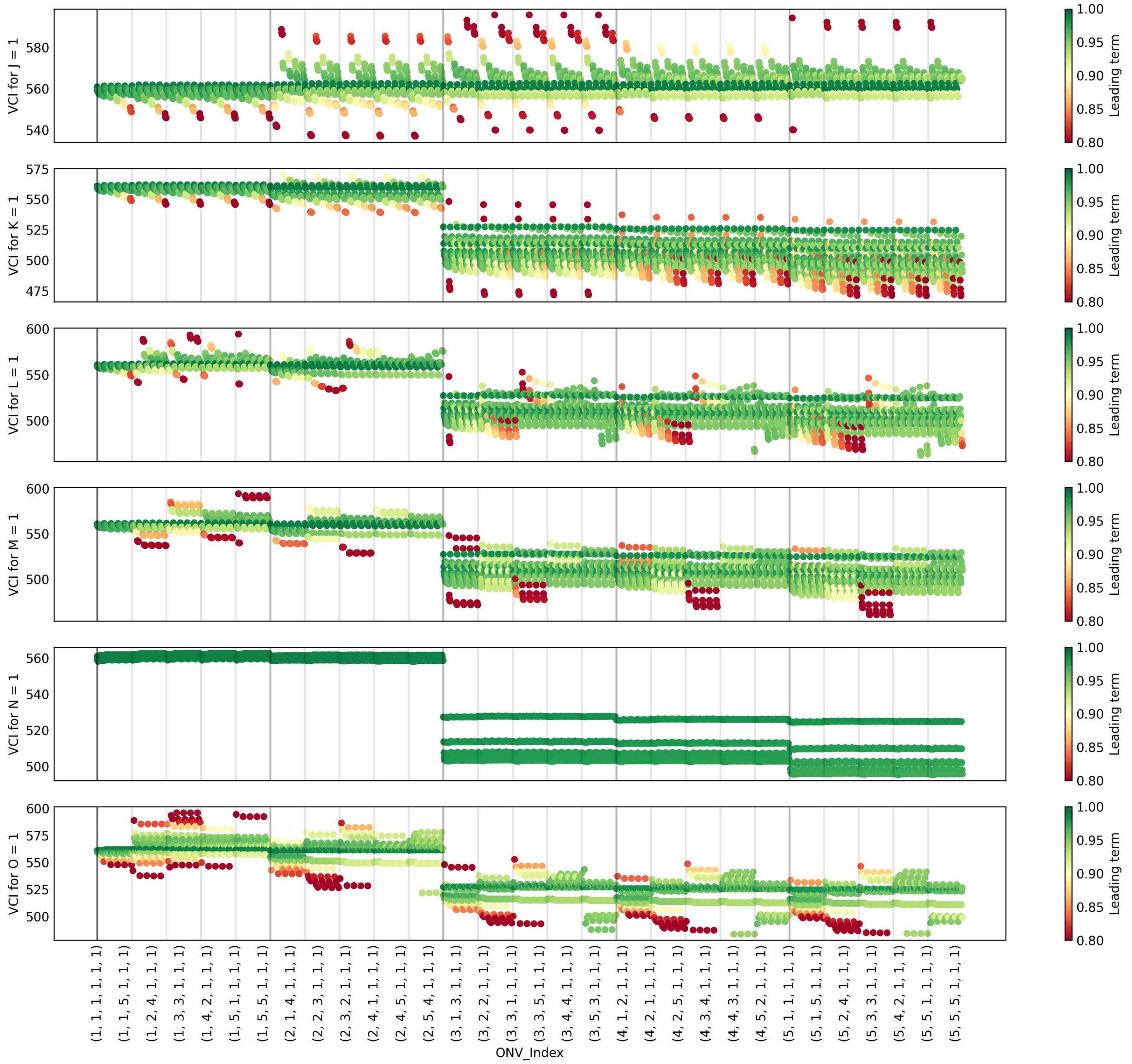

Figure S3.5: Benchmark for the VCI calculated fundamental transitions frequencies to $\nu_1(A')$ with respect to the maximum level of excitation in generating the VCI configurations. For each mode, we set `levex=1` and let the other modes vary. Reliable assignments are green.

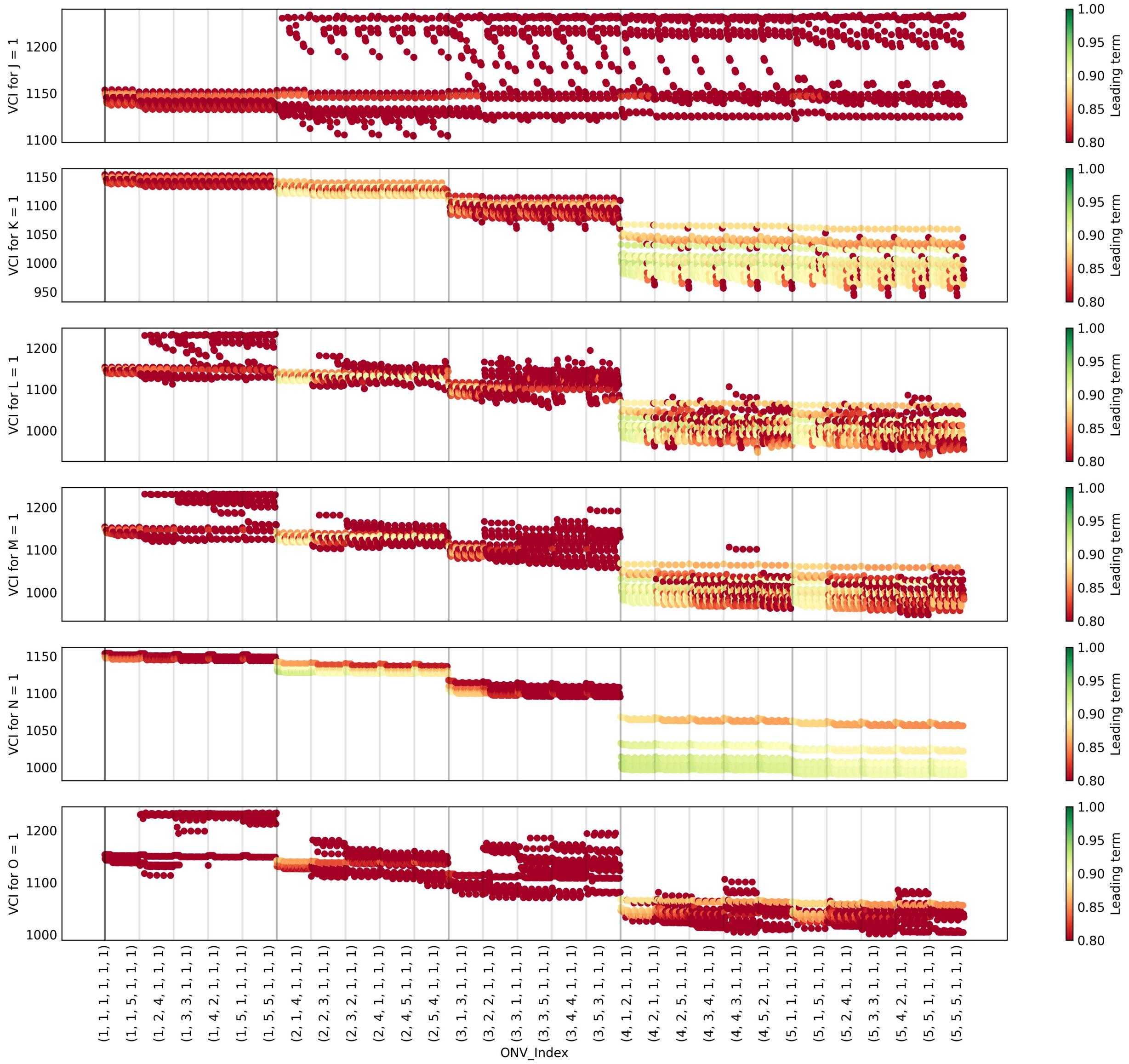

Figure S3.6: Benchmark for the VCI calculated fundamental transitions frequencies to $\nu_1^2(A')$ with respect to the maximum level of excitation in generating the VCI configurations. For each mode, we set `levex=1` and let the other modes vary. Reliable assignments are green.

S3.3 Harmonic & VSCF frequencies up to 5000 cm^{-1}

The following VSCF frequencies are not converged. This is mainly due to the problematic PES they are based on. As discussed in Section S3, the current multi-mode PES is troublesome due to the double-minimum subpotential of mode q_5. This is also reflected in the VSCF results presented here. All the calculations shown here are based on the 4-mode PES of $HOH \cdot H^-$ in a polynomial representation, where troublesome subpotentials have been excluded (delauto=1.d-5). We used the

PESTRANS utility to obtain mass weighted PESs for the various isotopomers. All calculations are cc-VSCF. These calculations are merely a test whether the isotopic transformation works, and as one may see from the following tables, it does for the harmonic frequencies, but the VSCF calculations again are not converged.

Table S3.2: $HOH \cdot H^-$ at CCSD(T)-F12/AVTZ-F12.

Mode Irrep	Harmonic	VSCF	Intensity
6^1 A'	3853.10	3660.16	4.38
5^1 A'	2821.63	2421.98	2901.14
4^1 A'	1679.30	1663.20	135.19
3^1 A"	942.82	1032.91	153.83
2^1 A'	639.94	657.80	1325.90
1^1 A'	495.01	657.94	198.64
4^2 A'	3358.60	3306.45	3.09
3^2 A'	1885.64	2115.36	0.99
2^2 A'	1279.87	1282.08	78.37
1^2 A'	990.03	1389.19	0.14
$3^1 + 6^1$ A"	4795.92	4700.44	0.05
$2^1 + 6^1$ A'	4493.04	4318.04	0.24
$1^1 + 6^1$ A'	4348.12	4370.02	5.93
$4^1 + 5^1$ A'	4500.93	4191.77	0.93
$3^1 + 5^1$ A"	3764.45	3372.28	2.92
$2^1 + 5^1$ A'	3461.57	2694.12	37.62
$1^1 + 5^1$ A'	3316.65	2922.81	24.38
$3^1 + 4^1$ A"	2622.12	2732.41	0.25
$2^1 + 4^1$ A'	2319.24	2312.50	2.79
$1^1 + 4^1$ A'	2174.31	2261.53	2.28
$2^1 + 3^1$ A"	1582.75	1743.12	1.47
$1^1 + 3^1$ A"	1437.83	1700.08	0.19
$1^1 + 2^1$ A'	1134.95	1319.45	2.77

Table S3.3: $HOH \cdot D^-$ at CCSD(T)-F12/AVTZ-F12.

Mode Irrep	Harmonic	VSCF	Intensity
6^1 A'	3853.10	3649.82	1.10
5^1 A'	2816.93	2241.54	711.92
4^1 A'	1673.68	1593.30	157.48
3^1 A"	911.30	992.75	131.29

(Table continues on next page)

Continuation of Table S3.9

Mode	Harmonic	VSCF	Intensity
2^1 A'	481.72	749.44	511.19
1^1 A'	451.25	716.89	436.14
4^2 A'	3347.37	3175.15	3.73
3^2 A'	1822.59	2055.24	0.98
2^2 A'	963.44	1518.87	0.29
1^2 A'	902.50	1428.23	6.94
3^1+6^1 A"	4764.39	4652.61	0.06
2^1+6^1 A'	4334.82	4425.68	2.18
1^1+6^1 A'	4304.35	4369.72	3.16
4^1+5^1 A'	4490.61	4332.26	0.02
3^1+5^1 A"	3728.22	3493.96	0.77
2^1+5^1 A'	3298.65	2056.99	0.49
1^1+5^1 A'	3268.18	2106.53	1.26
3^1+4^1 A"	2584.98	2629.53	0.25
2^1+4^1 A'	2155.40	2304.70	3.54
1^1+4^1 A'	2124.93	2291.77	0.67
2^1+3^1 A"	1393.02	1760.03	0.20
1^1+3^1 A"	1362.55	1724.33	0.74
1^1+2^1 A'	932.97	1437.19	0.90

Table S3.4: $HOD\cdot H^-$ at CCSD(T)-F12/AVTZ-F12.

Mode Irrep	Harmonic	VSCF	Intensity
6^1 A'	3851.75	3663.12	5.38
5^1 A'	2066.41	1922.20	1010.96
4^1 A'	1402.83	1401.07	84.04
3^1 A"	719.87	776.47	95.74
2^1 A'	634.71	632.82	1310.40
1^1 A'	448.31	584.51	172.62
5^2 A'	4132.82	3605.37	11.96
4^2 A'	2805.66	2791.53	2.68
3^2 A'	1439.74	1586.68	0.35
2^2 A'	1269.42	1233.21	69.06
1^2 A'	896.62	1229.42	0.10
3^1+6^1 A"	4571.62	4445.49	0.05
2^1+6^1 A'	4486.46	4294.86	0.20
1^1+6^1 A'	4300.06	4291.40	5.13

(Table continues on next page)

Continuation of Table S3.9

Mode	Harmonic	VSCF	Intensity
$4^1 + 5^1$ A'	3469.24	3382.24	0.08
$3^1 + 5^1$ A"	2786.28	2681.86	0.89
$2^1 + 5^1$ A'	2701.12	2420.13	0.41
$1^1 + 5^1$ A'	2514.72	2445.86	12.47
$3^1 + 4^1$ A"	2122.70	2207.13	0.08
$2^1 + 4^1$ A'	2037.54	2020.02	1.11
$1^1 + 4^1$ A'	1851.14	1911.45	0.82
$2^1 + 3^1$ A"	1354.58	1452.71	0.96
$1^1 + 3^1$ A"	1168.18	1374.51	0.11
$1^1 + 2^1$ A'	1083.02	1228.12	2.77

Table S3.5: $HOD \cdot D^-$ at CCSD(T)-F12/AVTZ-F12.

Mode Irrep	Harmonic	VSCF	Intensity
6^1 A'	3851.74	3789.58	33.43
5^1 A'	2059.11	23335.43	1.01
4^1 A'	1397.37	1084.68	157.33
3^1 A"	678.06	4030.58	187.44
2^1 A'	466.72	-10297.03	-0.08
1^1 A'	408.88	-440.52	-68.29
5^2 A'	4118.21	39779.64	0.49
4^2 A'	2794.75	2272.82	4.97
3^2 A'	1356.12	6822.48	3.98
2^2 A'	933.44	-9600.71	-0.71
1^2 A'	817.75	-1270.31	-0.65
$3^1 + 6^1$ A"	4529.80	7822.53	0.01
$2^1 + 6^1$ A'	4318.46	-6762.07	-0.00
$1^1 + 6^1$ A'	4260.62	3364.67	0.16
$4^1 + 5^1$ A'	3456.48	24345.51	0.00
$3^1 + 5^1$ A"	2737.17	25393.79	0.00
$2^1 + 5^1$ A'	2525.82	29519.52	0.00
$1^1 + 5^1$ A'	2467.98	24847.26	0.00
$3^1 + 4^1$ A"	2075.44	5056.33	0.23
$2^1 + 4^1$ A'	1864.09	-8763.94	-0.00
$1^1 + 4^1$ A'	1806.25	860.31	0.33
$2^1 + 3^1$ A"	1144.78	-6343.82	-0.00
$1^1 + 3^1$ A"	1086.94	3618.47	0.41
$1^1 + 2^1$ A'	875.60	-10459.45	-0.00

Table S3.6: $DOH \cdot H^-$ at CCSD(T)-F12/AVTZ-F12.

Mode	Irrep	Harmonic	VSCF	Intensity
6^1	A'	2845.64	2815.33	1724.67
5^1	A'	2776.63	2674.84	912.21
4^1	A'	1550.25	1535.24	91.98
3^1	A"	940.95	988.39	158.20
2^1	A'	636.01	611.62	1335.47
1^1	A'	412.39	493.09	142.55
4^2	A'	3100.50	3062.47	1.26
3^2	A'	1881.90	2035.31	0.91
2^2	A'	1272.03	1199.15	72.94
1^2	A'	824.77	1064.99	0.19
$4^1 + 6^1$	A'	4395.89	4391.27	0.85
$3^1 + 6^1$	A"	3786.59	3856.42	1.36
$2^1 + 6^1$	A'	3481.65	3307.82	38.54
$1^1 + 6^1$	A'	3258.03	3404.72	6.12
$4^1 + 5^1$	A'	4326.88	4227.48	1.41
$3^1 + 5^1$	A"	3717.58	3667.03	1.20
$2^1 + 5^1$	A'	3412.65	3231.95	22.77
$1^1 + 5^1$	A'	3189.02	3163.78	14.05
$3^1 + 4^1$	A"	2491.20	2540.14	0.23
$2^1 + 4^1$	A'	2186.27	2160.07	3.08
$1^1 + 4^1$	A'	1962.64	2002.73	2.22
$2^1 + 3^1$	A"	1576.96	1651.64	1.57
$1^1 + 3^1$	A"	1353.34	1479.85	0.07
$1^1 + 2^1$	A'	1048.40	1118.73	2.05

Table S3.7: $DOH \cdot D^-$ at CCSD(T)-F12/AVTZ-F12.

Mode	Irrep	Harmonic	VSCF	Intensity
6^1	A'	2842.84	2824.11	1829.25
5^1	A'	2774.71	2663.79	1114.75
4^1	A'	1542.91	1529.21	114.00
3^1	A"	909.36	959.06	143.66
2^1	A'	462.27	450.28	711.52
1^1	A'	382.01	451.47	117.59
4^2	A'	3085.82	3047.26	1.58
3^2	A'	1818.73	1970.04	0.68
2^2	A'	924.55	883.02	31.10

(Table continues on next page)

Continuation of Table S3.9

Mode	Harmonic	VSCF	Intensity
1^2 A'	764.02	973.43	0.11
4^1+6^1 A'	4385.75	4393.90	1.36
3^1+6^1 A"	3752.20	3835.18	1.34
2^1+6^1 A'	3305.11	3189.14	27.40
1^1+6^1 A'	3224.85	3364.03	5.38
4^1+5^1 A'	4317.62	4211.07	1.50
3^1+5^1 A"	3684.07	3626.16	1.34
2^1+5^1 A'	3236.98	3061.46	17.49
1^1+5^1 A'	3156.72	3100.86	14.02
3^1+4^1 A"	2452.27	2508.70	0.24
2^1+4^1 A'	2005.18	1986.44	2.13
1^1+4^1 A'	1924.92	1953.65	2.04
2^1+3^1 A"	1371.64	1444.26	0.97
1^1+3^1 A"	1291.37	1410.09	0.07
1^1+2^1 A'	844.28	912.44	1.17

Table S3.8: DOD $\cdot$ H^- at CCSD(T)-F12/AVTZ-F12.

Mode Irrep	Harmonic	VSCF	Intensity
6^1 A'	2801.98	2704.37	4.62
5^1 A'	2063.47	1935.56	1039.08
4^1 A'	1233.55	1220.92	23.19
3^1 A"	717.42	763.85	92.23
2^1 A'	629.90	631.15	1296.60
1^1 A'	391.05	500.56	133.41
5^2 A'	4126.94	3649.61	15.39
4^2 A'	2467.10	2434.48	0.88
3^2 A'	1434.85	1561.21	0.36
2^2 A'	1259.81	1230.80	73.97
1^2 A'	782.11	1056.96	0.10
5^1+6^1 A'	4865.45	4643.88	0.37
4^1+6^1 A'	4035.53	3939.74	0.06
3^1+6^1 A"	3519.40	3474.19	0.02
2^1+6^1 A'	3431.88	3330.44	0.25
1^1+6^1 A'	3193.03	3240.85	2.16
4^1+5^1 A'	3297.02	3203.03	0.02
3^1+5^1 A"	2780.89	2682.12	0.90

(Table continues on next page)

Continuation of Table S3.9

Mode	Harmonic	VSCF	Intensity
$2^1 + 5^1$ A'	2693.37	2412.60	0.33
$1^1 + 5^1$ A'	2454.52	2381.78	8.99
$3^1 + 4^1$ A"	1950.97	1998.76	0.05
$2^1 + 4^1$ A'	1863.45	1848.25	1.25
$1^1 + 4^1$ A'	1624.60	1687.20	1.23
$2^1 + 3^1$ A"	1347.33	1434.13	0.99
$1^1 + 3^1$ A"	1108.48	1268.02	0.05
$1^1 + 2^1$ A'	1020.96	1145.12	2.04

Table S3.9: $DOD \cdot D^-$ at CCSD(T)-F12/AVTZ-F12.

Mode Irrep	Harmonic	VSCF	Intensity
6^1 A'	2801.97	2706.97	9.43
5^1 A'	2056.12	-11843.78	-0.39
4^1 A'	1225.22	1292.99	46.74
3^1 A"	675.46	6042.72	527.17
2^1 A'	458.60	2537.48	854.04
1^1 A'	354.81	-81.67	-5.78
5^2 A'	4112.24	-10421.00	-0.19
4^2 A'	2450.44	2576.48	0.94
3^2 A'	1350.93	7525.28	9.05
2^2 A'	917.20	4288.69	12.52
1^2 A'	709.63	-441.28	-0.00
$5^1 + 6^1$ A'	4858.09	-9137.80	-0.00
$4^1 + 6^1$ A'	4027.20	4016.50	0.08
$3^1 + 6^1$ A"	3477.44	8757.12	0.03
$2^1 + 6^1$ A'	3260.57	5243.38	0.04
$1^1 + 6^1$ A'	3156.79	2638.51	0.28
$4^1 + 5^1$ A'	3281.34	-10619.85	-0.00
$3^1 + 5^1$ A"	2731.58	-9082.58	-0.00
$2^1 + 5^1$ A'	2514.72	-9475.27	-0.00
$1^1 + 5^1$ A'	2410.93	-10123.04	-0.00
$3^1 + 4^1$ A"	1900.68	7355.19	0.20
$2^1 + 4^1$ A'	1683.82	3830.98	0.43
$1^1 + 4^1$ A'	1580.03	1200.42	0.09
$2^1 + 3^1$ A"	1134.06	8586.20	0.47
$1^1 + 3^1$ A"	1030.28	5970.93	0.03
$1^1 + 2^1$ A'	813.41	2456.59	0.17

S4 Appendix

Table S4.1: 3-mode PES of (HOH)H- using CCSD(T)-F12/AVTZ-F12. The 1-mode potentials are shown as grid and polynomial representation delauto=off. The 3-mode PES is shown via effective potentials from ca-VSCF.

GRID (1D)	POLY (1D)	VSCF (3D)

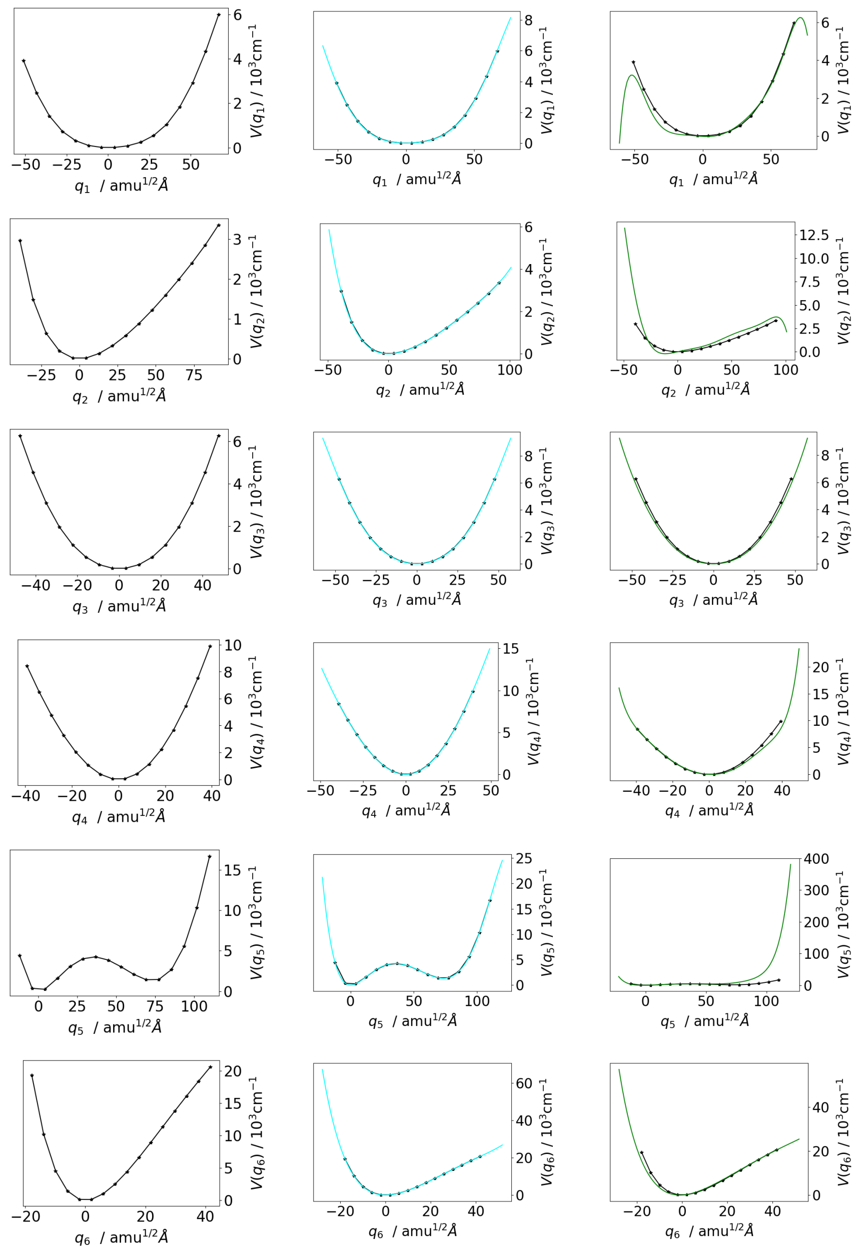

Table S4.2: Effective potentials of (HOH)H- from cc-VSCF using a polynomial representation of the multi-mode PES.

GRID (1D)	POLY (1D)	VSCF (3D)

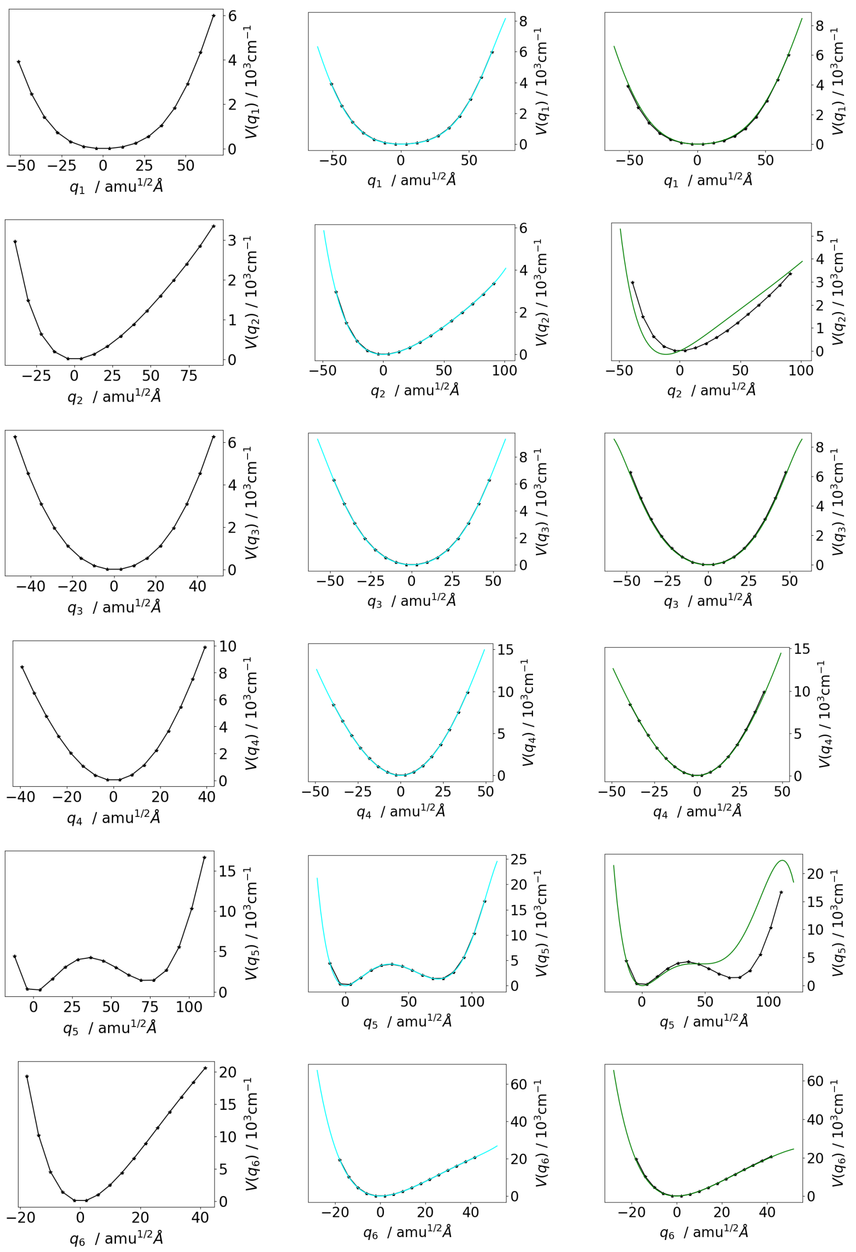

Table S4.3: Effective potentials of (HOH)H- from cc-VSCF using a polynomial representation of the multi-mode PES.

GRID (1D)	POLY (1D)	VSCF (3D)
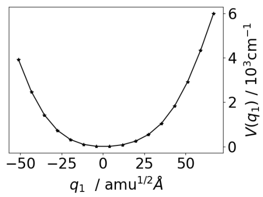	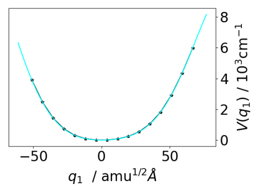	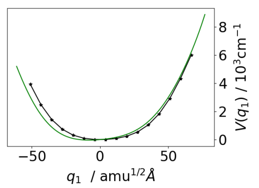
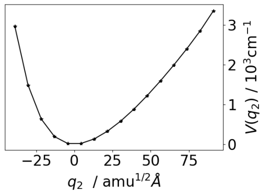	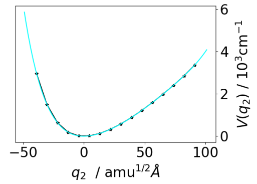	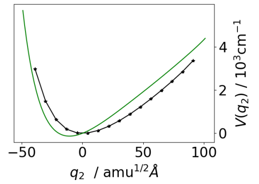
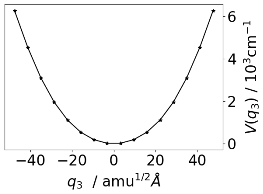	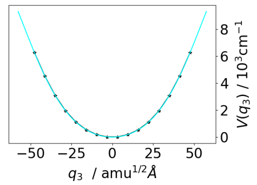	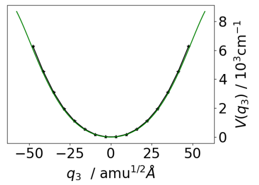
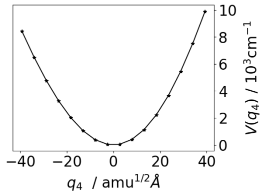	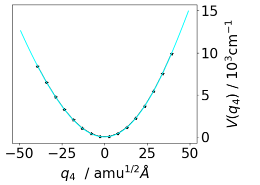	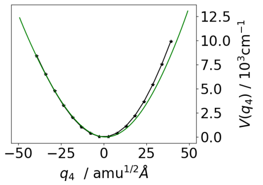
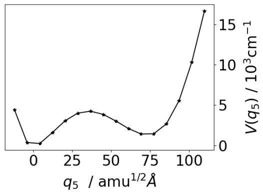	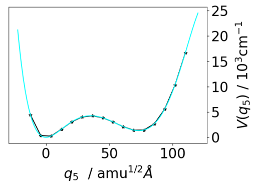	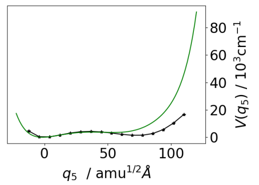
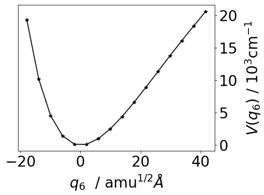	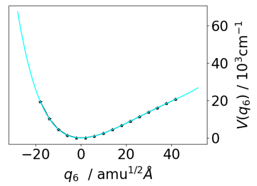	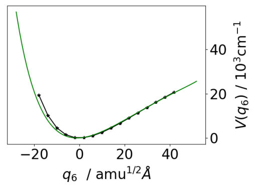
